# Dual-heterodyne Kelvin probe force microscopy

**DOI:** 10.3762/bjnano.14.88

**Published:** 2023-11-07

**Authors:** Benjamin Grévin, Fatima Husainy, Dmitry Aldakov, Cyril Aumaître

**Affiliations:** 1 Univ. Grenoble Alpes, CNRS, CEA, IRIG-SyMMES, 38000 Grenoble, France

**Keywords:** heterodyne, intermodulation, KPFM, nc-AFM, surface photovoltage, time-resolved measurements

## Abstract

We present a new open-loop implementation of Kelvin probe force microscopy (KPFM) that provides access to the Fourier spectrum of the time-periodic surface electrostatic potential generated under optical (or electrical) pumping with an atomic force microscope. The modulus and phase coefficients are probed by exploiting a double heterodyne frequency mixing effect between the mechanical oscillation of the cantilever, modulated components of the time-periodic electrostatic potential at harmonic frequencies of the pump, and an ac bias modulation signal. Each harmonic can be selectively transferred to the second cantilever eigenmode. We show how phase coherent sideband generation and signal demodulation at the second eigenmode can be achieved by using two numerical lock-in amplifiers configured in cascade. Dual-heterodyne KPFM (DHe-KPFM) can be used to map any harmonic (amplitude/phase) of the time-periodic surface potential at a standard scanning speed. The Fourier spectrum (series of harmonics) can also be recorded in spectroscopic mode (DHe-KPFM spectroscopy), and 2D dynamic images can be acquired in data cube mode. The capabilities of DHe-KPFM in terms of time-resolved measurements, surface photovoltage (SPV) imaging, and detection of weak SPV signals are demonstrated through a series of experiments on difference surfaces: a reference substrate, a bulk organic photovoltaic heterojunction thin film, and an optoelectronic interface obtained by depositing caesium lead bromide perovskite nanosheets on a graphite surface. The conclusion provides perspectives for future improvements and applications.

## Introduction

Kelvin probe force microscopy (KPFM) is a well-known variant of AFM that allows probing at the nanoscale the electrostatic landscape on the surface of a sample by measuring the so-called contact potential difference (CPD). If one considers a simple junction formed by a metallic AFM tip and a metallic sample, the CPD originates from the tip–sample work function difference. More generally, the CPD stems from the existence of electric charges and/or dipoles in the system under consideration. As a result, the applications of KPFM are extremely broad. It is now used by physicists, chemists, and biologists to characterize the nanoscale electronic/electrostatic properties of an ever-expanding range of materials, interfaces, and devices, in ambient conditions, under ultrahigh vacuum, or at the liquid–substrate interface.

Since the early 90’s, a variety of approaches have been implemented to improve KPFM performances in terms of spatial, potentiometric, and temporal resolution. Several research teams continue to work in this direction and KPFM is still an evolving technique in many aspects. In particular, the development of KPFM-based approaches specifically designed to investigate photogeneration mechanisms and charge dynamics at the nanoscale in photovoltaic and optoelectronic materials is an active research area.

In photoassisted KPFM, the idea is to probe the surface photovoltage (SPV), which is the illumination-induced change in the surface electrostatic potential (i.e., the opposite of the CPD shift under illumination). To map the SPV, the most basic approach consists in performing a dual-pass experiment. Two CPD maps are recorded, the first in the “dark state” and the second under continuous wave (cw) illumination. An SPV image can eventually be recalculated as the difference between both sets of data [1]. The inherent drawback of this dual-pass method is that the source images used for the SPV calculation may feature misalignments, which is especially unavoidable at room temperature (RT) due to thermal drift [2–3]. Another issue is that some of the SPV components – related to dynamical processes – can remain hidden to this “conventional” SPV imaging [4].

To mitigate the effects of thermal drift, a first alternative consists in performing a data cube acquisition of CPD curves synchronously recorded with the application of a light pulse (i.e., a curve is recorded at each point of the surface along a 2D grid). Again, a differential SPV image can be reconstructed from the matrix of spectroscopic curves [5]. However, this approach is not free from artefacts, in particular the cantilever photothermal bending can significantly modify (depending on the optical power) the tip–surface distance during the spectroscopic measurement (which has to be performed in open z-loop).

Whenever possible, it is preferable to investigate the photoresponse of the sample under modulated or pulsed illumination. Provided that the modulation frequency of the light source is set high enough, it becomes possible to “stabilize” the static bending in a steady state, and photothermal cantilever excitation can be avoided (by setting the optical modulation at a frequency that differs from the cantilever resonance eigenmodes).

Working under modulated illumination also allows direct SPV measurements using special modulation/demodulation schemes, such as the one recently proposed by Miyazaki and co-workers for ac-bias KPFM [6]. In addition, most time-resolved KPFM modes developed so far rely on the use of a pulsed/modulated illumination chain. This is for instance the case of intensity-modulated KPFM [7–9], pump-probe KPFM [4,10–11], or G-mode KPFM [12], to name a few (we refer the readers to review articles for a more comprehensive introduction to AFM-based time-resolved potentiometric and electrostatic modes [13–14]).

Each of these approaches have advantages and disadvantages. Intensity-modulated KPFM implementation is straightforward, since it simply consists in analysing the frequency response of the time-average CPD under modulated illumination. Nevertheless, it is prone to capacitive artefacts [15]. Accurate quantitative measurements can be performed by pump-probe KPFM (pp-KPFM), with a time-resolution down to the sub-nanosecond scale [10]. However, pp-KPFM features a severely limited bandwidth. In the case of our last implementation of pp-KPFM [4], several tens of seconds (to minutes) are needed to record a single spectroscopic curve, and tens of hours to several days are needed to map a 2D matrix of data. Last, G-mode KPFM requires analysis of large datasets acquired with high-speed data capture [12].

A few years ago, Borgani and Haviland presented an interesting alternative for time-resolved SPV measurements [16], which consists in analysing the intermodulation products between the mechanical oscillation of the cantilever and the photogenerated surface potential. In short, intermodulation spectroscopy allows working in the frequency domain (instead of the time domain) by extracting, during a single measurement, quantities that are directly proportional to the Fourier coefficients of the time-periodic surface photovoltage. Unfortunately, the translation of this promising technique from ambient conditions – where it has proven its worth – to ultra-high vacuum poses significant challenges. This is mainly due to the enhanced quality factors under vacuum, which severely limits the frequency window available to increase the amplitude of the intermodulation products (for a more detailed discussion, see [14]).

In this work, we propose to approach the measurement of intermodulation products with non-contact AFM under ultra-high vacuum in a slightly different way. We demonstrate that the Fourier spectrum (modulus and phase coefficients) of the time-periodic electrostatic surface potential generated under optical (or electrical) pumping can be probed, by exploiting a double heterodyne frequency mixing effect between the cantilever mechanical oscillation, the surface photovoltage harmonics, and an ac bias modulation signal. The frequency of the modulated bias can be set so that any given spectral component of the surface potential (or intermodulation product) can be “transferred” to the second cantilever eigenmode. This transfer or rejection (i.e., a measurement of a Fourier harmonic at the pump frequency through a demodulation at the second eigenmode frequency) is achieved by using two numerical lock-in amplifiers in cascade, which allows the generation of proper phase-coherent combination of reference signals.

Dual-heterodyne KPFM (DHe-KPFM) dramatically enhances the sensitivity to “weak” (i.e., a few mV) surface photovoltage signals, thanks to the amplification by the second resonance mode. Time-resolved measurements can be performed in data-cube mode, by recording spectra of the SPV Fourier components (modulus and phase) onto a 2D grid. The validity of DHe-KPFM implementation is first demonstrated by carrying out measurements on a conducting reference substrate under electrical pumping. The results confirm that – thanks to the constant transfer function over the entire spectrum provided by the signal demodulation at the second cantilever eigenmode – a quantitative measurement of the Fourier spectrum can be performed. In the case of the amplitude spectrum, that measurement is done within a scalar factor related to the capacitance gradient. Furthermore, 2D images of time constant that characterize the surface photovoltage dynamics are acquired on a photovoltaic organic bulk-heterojunction (BHJ) thin film. The time-constant values are shown to be fully consistent with the results of pump-probe KPFM. Last, the ability of DHe-KPFM to detect weak SPV signals is illustrated by investigating optoelectronic interfaces formed between caesium lead bromide perovskite nanosheets and highly oriented pyrolytic graphite.

## Kelvin Probe Force Microscopy Background, Amplitude-Modulated Heterodyne KPFM

Many KPFM modes rely on the detection of a modulated component of the electrostatic force proportional to the CPD, using a lock-in amplifier. The attractive electrostatic force between the tip and the surface can be expressed as:


[1] $$F_{\text{el}}=\frac{1}{2}{C^{\prime }}_{\text{z}}{\left(V_{\text{bias}}\pm V_{\text{CPD}}\right)}^{2}.$$

In the above equation, *C'*_z_ stands for the tip–sample capacitive gradient. *V*_bias_ represents the external bias voltage applied to the system (including both a dc and an ac component, see hereafter). The plus or minus sign applies if *V*_bias_ is applied to the tip (+ sign) or to the sample (− sign), respectively.

The lock-in output provides a modulated ac bias voltage (*V*_ac_, amplitude *U*_ac_, and angular frequency ω_ac_) that is added to the static dc voltage applied to the tip (or sample), *V*_bias_ = *V*_ac_ + *V*_dc_. The electrostatic force displays modulated components, and some are directly proportional to the dc electrostatic potential term (i.e., *V*_dc_ ± *V*_CPD_). The idea is to demodulate such a component, and feed it into a loop which controls the dc bias. The KPFM controller (with a zero set point), will eventually adjusts the dc bias to minimize its input, yielding a measurement of the CPD (*V*_dc_ = ±*V*_CPD_ depending on the setup geometry). In the following, the dc compensation bias provided to the tip by the KPFM controller (in our setup *V*_dc_ = −CPD) will be referred to as either *V*_dc_ or KPFM potential. To avoid repetition, this quantity is sometimes also referred to as "surface potential". This is obviously a misuse of language, as KPFM gives a relative measurement of the surface electrostatic potential with respect to that of the tip. However, it is partly justified because in our setup the compensation bias is applied to the tip. Then, provided that the effective work function of the tip remains constant during the measurement, any change in the KPFM compensation potential will strictly follow the change in the surface electrostatic potential without sign inversion (a local positive surface charging corresponds to a positive *V*_dc_ change).

Actually, there are many ways to perform the signal demodulation. Amplitude heterodyne KPFM, introduced in 2012 by Sugawara et al. [17], is a very elegant approach. It combines the advantages of frequency-modulated KPFM (FM-KPFM) and amplitude-modulated KPFM (in terms of lateral resolution and sensitivity, respectively). This mode takes advantage of heterodyning effects (frequency mixing) between the electrical bias modulation and the cantilever mechanical oscillation (usually performed at the first eigenmode, angular frequency ω_0_). These effects result in the existence of additional modulated component of the electrostatic force (or sidebands) at frequencies ω_0_ ± ω_ac_. They stem from higher harmonic components in the capacitance term [16,18]:


[2] $${C^{\prime }}_{\text{z}}=K_{0}+\sum_{n=1}^{+\infty }K_{n}\cos\left(n\omega_{0}t\right)=K_{0}+K_{1}\cos\left(\omega_{0}t\right)+...$$

If we restrict ourselves to the first harmonic (*n* = 1), electrostatic force components emerge (we just describe the first set of sidebands, other exist at ω_0_ ± 2ω_ac_) which are:


[3]

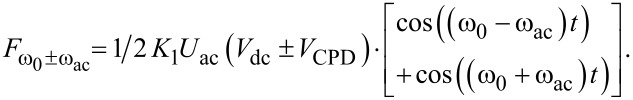



It can be shown [18], that the Fourier coefficient at *n* = 1 is proportional to the second z-derivative of the capacitance (*K*_1_ ≡ *C''*_z_). Consequently, demodulating the amplitude of the sidebands gives access at a term that is proportional to the dc potential difference, and one works (like in frequency-modulation KPFM) on a signal that is proportional to the electrostatic force gradient (through *C''*_z_). For that reason, this “side-band” KPFM is often referred to as a variant of FM-KPFM.

Amplitude-modulated heterodyne KPFM [17] further improves this approach. The idea is to set the modulated bias angular frequency so that the first right sideband (ω_0_ + ω_ac_) is “rejected” at the second mechanical resonance eigenmode (frequency ω_1_) of the cantilever. Implementing this modulation/demodulation scheme is nowadays greatly facilitated by the flexibility offered by last-generation digital lock-in amplifiers, which allow generating (and demodulating) phase-coherent combinations of multiple oscillators (i.e., an excitation at ω_ac_ = ω_1_ − ω_0_ and a demodulation at ω_1_).

Here, it is mandatory to stress a very important point. In all strictness, in the above, we should have written that ω_ac_ = ω_1_ − (ω_0_ + Δω_0_), where Δω_0_ stands for the cantilever frequency shift due to the tip–surface interaction. In heterodyne KPFM, the reference sideband that drives the modulated bias is indeed generated as follows. The frequency of the first source signal (or lock-in internal oscillator) is set to ω_1_, ultimately it will be used for signal demodulation. The second source signal is obtained by tracking the cantilever resonance frequency at its first eigenmode (ω_0_ + Δω_0_) with a second oscillator configured as a phase-locked loop (PLL). This tracking is necessary because the first mode is used by the main AFM controller for topographic control (in frequency modulation mode in the case of non-contact AFM under UHV). In other words, it is the AFM controller which generates the source signal at ω_0_ + Δω_0_ which “excites” the mechanical oscillation of the cantilever. The frequency mixing effect will effectively generate a modulated electrostatic component at ω_1_ – phase-coherent with the demodulation chain – only and if only the frequency shift that the cantilever (at its first resonance mode) experiences in the tip–surface interaction is taken into account. In FM-AFM, it is impossible to perform a heterodyne-KPFM measurement by setting a “fixed” value for ω_0_.

## Dual-Heterodyne Kelvin Probe Force Microscopy: Principle

To understand how dual-heterodyne KPFM works, consider the case of a sample subjected to a time-periodic pump that generates a (time-)periodic electrostatic potential. Within the framework of investigations on photoactive materials, of course, the pump will consist of light pulses (in which case one generates a periodic SPV), but DHe KPFM experiments (as shown below) can also be performed under electrical pumping (by applying bias pulses directly to the sample with an arbitrary waveform generator).

The time-periodic electrostatic potential *V*_mod_ (or photopotential) generated at the sample under modulated illumination can be described by a Fourier series:


[4] $$V_{\mathrm{mod}}=V_{0}+\sum_{n=1}^{+\infty }V_{n}\cos\left(n\omega_{\mathrm{mod}}t+\phi_{n}\right)$$

As in other KPFM modes, a combination of dc (*V*_dc_) and ac (*V*_ac_) bias is applied to the AFM tip. The total electrostatic force between the tip and the sample is now:


[5] $$\begin{array}{l} F_{\text{el}}=\frac{1}{2}{C^{\prime }}_{z}{\left[\left(V_{\text{dc}}+V_{\text{CPD}}\right)+V_{\text{ac}}-V_{\mathrm{mod}}\right]}^{2}\\ \text{with }V_{\text{ac}}=U_{\text{ac}}\cos\left(\omega_{\text{ac}}t\right) \end{array}$$

Note that in the above, we assumed that the dc potential is applied to the tip (this actually corresponds to the configuration of our setup). *V*_CPD_ encompasses the "in-dark" electrostatic potential difference between the tip and the sample. The additional time-averaged shift due to the SPV is accounted for by the first term in the Fourier series (*V*_0_). The harmonic terms (*n* ≥ 1 in Equation 4) contain all the information about the time dependence of the SPV. To prevent misunderstanding, it is important to stress that the amplitude spectrum (i.e., the *V*_n_ terms) should not be confused with the amplitude of the time-periodic SPV. Strictly speaking, the *V*_n_ terms correspond to the modulus of complex Fourier coefficients (see Supporting Information File 1). The reader is asked to keep this fact in mind when the *V*_n_ series will be referred to as the amplitude (modulus) spectrum in the following. Obviously, by linearity of the Fourier transform, each component of the “amplitude spectrum” (including the static term) is proportional to the amplitude (or magnitude) of the SPV. We will see later on how static (SPV magnitude) and dynamic (SPV decay time constant) images can be mapped by appropriately fitting the amplitude spectrum.

As with AM-heterodyne KPFM, there is a frequency mixing effect due to the capacitance term, and we restrict ourselves to the first harmonic to describe the time response of the capacitance gradient (*K*_1_, Equation 2). Moreover, under periodic pumping, an additional frequency mixing process occurs due to the simultaneous existence of modulated potentials at ω = ω_ac_ and ω = *n*ω_mod_. In our case, the terms of interest are those resulting from the multiplication of *V*_ac_ by *V*_mod_ in Equation 5 (in the following we will omit the other terms, bearing in mind that they exist):


[6] $$2U_{\text{ac}}\cos\left(\omega_{\text{ac}}t\right)\times \sum_{n=1}^{+\infty }V_{n}\cos\left(n\omega_{\mathrm{mod}}t+\phi_{n}\right).$$

This can be rewritten as:


[7] $$U_{\text{ac}}\sum_{n=1}^{+\infty }\begin{array}{l} \;V_{n}\cdot \cos\left(\left(n\omega_{\mathrm{mod}}+\omega_{\text{ac}}\right)t+\phi_{n}\right)\\ \;+V_{n}\cdot \cos\left(\left(n\omega_{\mathrm{mod}}-\omega_{\text{ac}}\right)t+\phi_{n}\right) \end{array}.$$

Finally, the other frequency mixing process (multiplication by the capacitive term, first harmonic) results in the appearance of additional spectral components in the oscillating electrostatic force, but this time there are 4 × *n* of them (instead of two sidebands for conventional AM-heterodyne KPFM):


[8] $$\frac{1}{4}K_{1}U_{\text{ac}}\sum_{n=1}^{+\infty }\begin{array}{l} V_{n}\cdot \cos\left(\left(\omega_{0}+n\omega_{\mathrm{mod}}+\omega_{\text{ac}}\right)t+\phi_{n}\right)\\ +V_{n}\cdot \cos\left(\left(\omega_{0}-n\omega_{\mathrm{mod}}-\omega_{\text{ac}}\right)t-\phi_{n}\right)\\ +V_{n}\cdot \cos\left(\left(\omega_{0}+n\omega_{\mathrm{mod}}-\omega_{\text{ac}}\right)t+\phi_{n}\right)\\ +V_{n}\cdot \cos\left(\left(\omega_{0}-n\omega_{\mathrm{mod}}+\omega_{\text{ac}}\right)t-\phi_{n}\right) \end{array}.$$

To avoid any confusion, in the following, the sidebands will be labelled as follow:


[9]

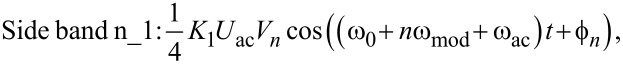




[10]

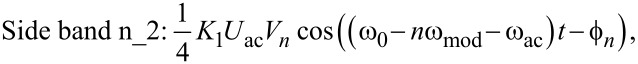




[11]

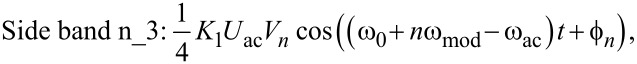




[12]

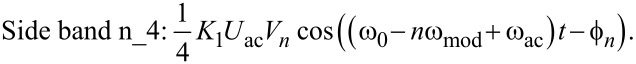



As with conventional AM-heterodyne KPFM, the idea is to use the second mechanical resonance eigenmode of the cantilever to "boost" a selected sideband. For example, the ac value can be adjusted so that the n_4 sideband frequency is rejected at the second eigenmode (i.e., ω_ac_ = ω_1_ – ω_0_ + *n*ω_mod_). Note that depending on the frequency range of the harmonics, other bands can be used for rejection (such as the n_1 one, with ω_ac_ = ω_1_ – ω_0_ – *n*ω_mod_).

Before going further, we would like to note that the same result (i.e., Equation 8 and Equations 9–12) would have been obtained by first considering a frequency mixing effect between the mechanical oscillation at ω_0_ and the modulated components at *n*ω_mod_, and then a second heterodyning process with the ac bias modulation. This may sound trivial, but considering things in this order may provide a useful way to visualise how harmonics can be “transferred” by properly setting the ac bias modulation frequency at the second eigenmode, as shown in Supporting Information File 1, Figure S4.

**Figure 1:**
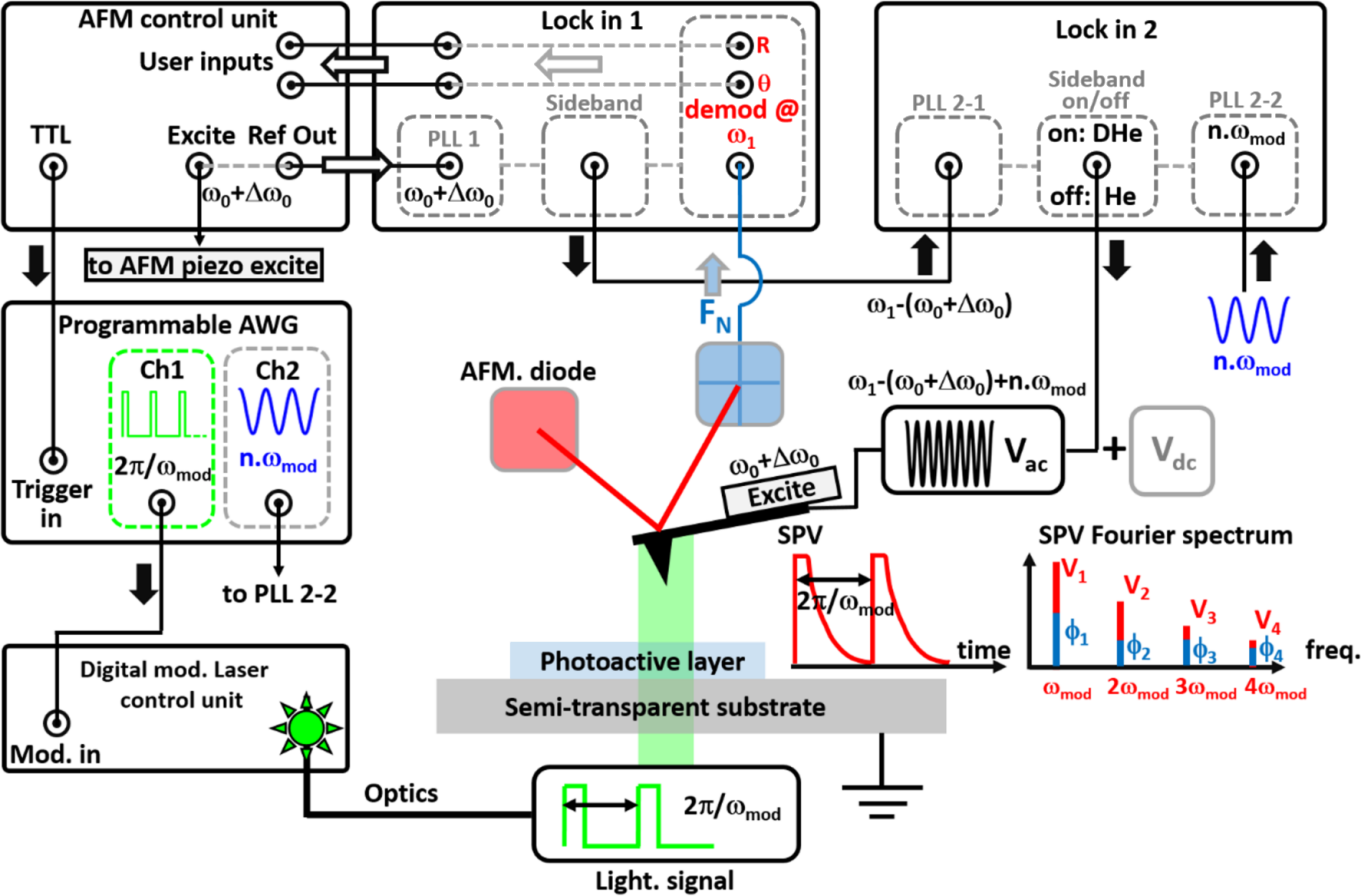
The implementation of DHe-KPFM. The real geometry of the sample stage (backside or front side illumination depending on the nature of the substrate) and the external optics differ from that of the simplified scheme. The cantilever is mechanically excited by the AFM control unit at its first eigenmode resonance frequency ω_0_, shifted by Δω_0_ in the tip-surface interaction. The first lock-in unit performs a combination (or sideband generation) to generate a reference sine at ω_1_ − (ω_0_ + Δω_0_). The "intermediate" reference signal at ω_1_ − (ω_0_ + Δω_0_) is subsequently fed to the external input of a second digital lock-in. The second lock-in combines it with another reference sine signal generated by the arbitrary waveform generator (AWG, channel 2) at *n*ω_mod_. Then, a modulated bias *V*_ac_ with an angular frequency ω_ac_ = ω_1_ – (ω_0_ + Δω_0_) + *n*ω_mod_ is generated at the lock-in output and applied to the AFM cantilever. NB: the configuration can be switched (software-controlled toggle of the modulation unit) between DHe-KPFM (DHe) and standard AM-heterodyne-KPFM (He). The AWG also generates the pump source signal (channel 1, i.e.; a square-waved modulation with period 2π/ω_mod_, duty ratio fixed at the user convenience). The time-periodic SPV displays multispectral components at the frequencies *n*ω_mod_ which stem from the dynamics of photoexcited states. Demodulating the normal force at ω_1_ yields access to the SPV *n*-th Fourier coefficients (amplitude and phase). Curves of the SPV Fourier spectrum are acquired by sequentially sweeping the output signal from the second channel of the AWG (i.e., *n*ω_mod_ with *n* = 1, *n* = 2, *n* = 3, etc.). This is achieved by playing a pre-programmed list of waveforms, triggered by TTL pulses generated by the AFM controller.

Demodulating the amplitude of the second eigenmode (at ω_1_) gives thus a direct access to the Fourier coefficients characterizing the *n*-th harmonic component of the time-periodic electrostatic potential of interest. It is important to understand that, in practice, such demodulation can only be carried out if phase (time) coherence is maintained between all the signals throughout the modulation/demodulation chain. This requires the use of two numerical lock-in amplifiers in cascade, as shown in Figure 1.

As with AM-heterodyne KPFM, the first lock-in unit performs a combination of two signals to generate a reference at the frequency ω_ref_ = ω_1_ − (ω_0_ + Δω_0_). The frequency of the first source signal (or internal oscillator) is set to ω_1_, ultimately it will be used for signal demodulation. The second source signal is obtained by tracking the cantilever resonance frequency at its first eigenmode (ω_0_ + Δω_0_) with a second oscillator configured in a PLL configuration.

In conventional AM-heterodyne KPFM, the reference signal at ω_1_ − (ω_0_ + Δω_0_) is used to perform the electrical excitation. In dual-heterodyne KPFM, it is instead used as an "intermediate" reference signal. It is subsequently fed to the external input of a second digital lock-in, which tracks it with a dedicated PLL. A second internal PLL is used to track a second reference signal generated by an arbitrary waveform generator at *n*ω_mod_. Actually, this AWG unit generates two in-phase coherent signals. The first channel generates a square-waved modulation (period 2π/ω_mod_, duty ratio fixed at the user convenience), which is used to pump the sample (via a digital modulated laser source, or a direct electrical connection to the sample for tests under electrical pumping). The second channel generates a sinusoidal reference signal at *n*ω_mod_. The modulated bias voltage *V*_ac_ can then be generated by the second digital lock-in by performing a phase-coherent combination of the two reference signals at ω_1_ − (ω_0_ + Δω_0_) and *n*ω_mod_. Finally, the amplitude and phase of the second eigenmode are demodulated at ω_1_ by the first lock-in unit. The schematic diagram in Figure 1 shows how the modulation/demodulation chain is set up.

To sum up, DHe-KPFM allows a selective demodulation of the Fourier coefficients (*V_n_*,Φ*_n_*) of the periodic electrostatic potential generated at the sample surface under a modulated pump.

The first and most straightforward application consists in mapping a selected component (such as the first harmonic *n* = 1) to obtain a set of images (amplitude/phase) directly related to the SPV generated under modulated/pulsed illumination. Series of images can be acquired for different pump parameters (period and duty ratio), providing a first approach to check the nature of the SPV dynamics. Compared to previous “modulated” SPV imaging techniques, dual-heterodyne KPFM provides an enhanced sensitivity (as demonstrated in the following), thanks to the amplification of the intermodulation products by the second resonance eigenmode.

In addition, alike intermodulation spectroscopy [16], time-resolved measurements can be performed by DHe-KPFM by recording several harmonics of the SPV signal. To that end, we implemented (as detailed hereafter in the “DHe-KPFM implementation” section) a spectroscopic variant which allows recording onto 2D grid curves of the Fourier coefficients (*V_n_*,Φ*_n_*) as a function of the harmonic number.

As far as the phase is concerned, two points should be stressed. The first is trivial: depending on the sideband used for rejection, a + or − sign must be applied after demodulating the signal (intermodulation products have a phase of ±Φ*_n_*, see Equation 8). More importantly, the phase measurement is relative, since the demodulation chain introduces an arbitrary phase shift with respect to the "zero" phase of the average component of the modulated SPV (*V*_0_ in Equation 4). To solve that issue, we added a refinement which consists in “switching” (during a selected time window of the spectroscopic sequence) the sideband generation to the standard AM-heterodyne KPFM scheme (i.e., ω_ac_ = ω_1_ – (ω_0_ + Δω_0_) instead of ω_ac_ = ω_1_ – (ω_0_ + Δω_0_) + *n*ω_mod_ when working with the 4*n*-th sidebands). In this configuration, the demodulated component yield access to the “dc” Fourier coefficient *V*_0_ and to the reference phase shift Φ_0_.

Compared to intermodulation spectroscopy [16], our approach has the disadvantage of being "sequential" in the sense that the Fourier components are measured one after the other and not during the same acquisition time window. However, it offers interesting advantages. First, the sideband rejection at the second eigenmode ensures that the transfer function remains exactly the same regardless of the measured harmonic. This allows a quantitative measurement of the Fourier components without the need for cantilever transfer function calibration. More importantly, there is virtually no limit to the number of harmonics that can be probed and the time resolution, other than that imposed by the maximum modulation/demodulation frequencies allowed by the lock-in units and the AWG. Last but not least, DHe-KPFM can be implemented on a non-contact AFM operating under ultra-high vacuum, where the high resonance quality factors should now become an advantage and provide greater sensitivity. In the next section, we will provide some details on the technical implementation of DHe-KPFM on an nc-AFM. We will then demonstrate its ability to detect weak SPV signals and perform time-resolved measurements.

Before ending this section, two final remarks should be made. DHe-KPFM is an "open loop" variant of KPFM. It does not rely on the application of an "active" compensation bias to probe the electrostatic potential of the sample. It yields a measurement of the Fourier spectrum of the time-periodic surface potential, which may be in principle used to reconstruct the signal in the time domain. However, in the current version of our implementation, this retro-conversion process from the frequency domain will only provide data that are proportional to the surface potential (or photopotential). This stems from the fact that the tip–sample capacitance second derivative *C''*_z_ (*K*_1_ term in Equation 8 and Equations 9–12) remains unknown. In principle, alike in the case of dual-harmonic KPFM [19], it should be possible to perform a quantitative measurement of *C''*_z_ by employing a complementary demodulation scheme. For that purpose, one would need to reject at the second eigenmode another set of sidebands (ω_0_ ± 2ω_ac_). This point – beyond the scope of the current study – will be the subject of a forthcoming work. In the remainder of this article, for the sake of brevity, we will omit to systematically recall that the measurement of the amplitude coefficients is performed within a multiplication by the factor *K*_1_. We will write *V_n_* instead of *K*_1_ × *V_n_*. The amplitude spectrum data will be given in mV (instead of arbitrary units), as they correspond to the demodulated amplitudes at the output of the locking device. However, once again, a confusion should not be made between these amplitudes (modulus) values and the SPV amplitude itself.

The second comment is also related to the fact that DHe-KPFM is an open variant of KPFM. It cannot be used for an active electrostatic compensation. However, as in the case of pump-probe KPFM [4,10], it is possible to use a second additional FM-KPFM loop which cancels the "time-averaged" dc part of the potential. This feature will not be used in this work, but its implementation has been validated. In the schematic (Figure 1), the dc bias applied to the tip represents the compensation bias of this second loop (it also has its own modulated bias, which is not shown in the schematic). As with pump-probe KPFM, there is no crosstalk between the two modulation/demodulation units because their operating frequency ranges are far apart. In FM-KPFM, the bias is modulated at a few kHz, while in the double-heterodyne scheme, much higher frequencies are used for electrical excitation. This spectral range difference also holds true for signal demodulation, which is performed at a few kHz for the FM-KPFM loop and hundreds of kHz (demodulation at the second eigenmode) for its heterodyne counterpart.

## Dual-Heterodyne Kelvin Probe Force Microscopy: Implementation

Non-contact AFM (nc-AFM) and KPFM experiments are performed with a VT-beam AFM system (Scienta-Omicron) at room temperature (RT) under ultra-high vacuum (UHV), driven by a Mimea scanning probe microscope (SPM) controller (SPECS-Nanonis). Topographic imaging is performed in FM mode (FM-AFM) in the attractive regime, with negative frequency shifts of a few Hz and vibration amplitudes of a few tens of nm. All experiments were performed with Pt/Ir coated silicon cantilevers (PPP-EFM, Nanosensors, resonance frequency in the range 45–115 kHz), annealed in situ to remove atmospheric contaminants.

The dual-heterodyne KPFM mode was implemented by combining the SPM unit with two digital lock-ins from Zurich Instruments (lock-in 1: MFLI, lock-in 2: HF2LI). Both are equipped with multiple oscillators, control loops, PLLs, and modulation modules (2 for the HF2LI). The MFLI modulation module is used to generate the intermediate reference signal with an angular frequency of ω_1_ – (ω_0_ + Δω_0_). The reference sidebands for the electrical excitation (*V*_ac_, ω_ac_) are generated by the HF2LI modulation module. As aforementioned, different series of sidebands can be used to probe the signal Fourier spectrum. In this regard, it should be noted that a 180 phase shift is added by the HF2LI to its output (*V*_ac_) if one uses the left sideband (with respect to the carrier, as defined in the HF2LI modulation module) instead of the right one. In our case, this additional 180 phase shift is taken into account when the n_1 sidebands are used instead of the n_4 ones.

The pump (modulated square wave with an adjustable duty ratio and period 2π/ω_mod_) and the harmonic reference (sine, angular freq. *n* × ω_mod_) are generated by a Keysight 33600 A arbitrary waveform generator. The maximum frequency for the sine signal is 120 MHz, 50 MHz for a square wave with a duty ratio of 25%. On its part, the HF2LI unit can handle signals up to 50 MHz. In principle, these combined features allow up to approximately 10 harmonics to be sampled with a base pump period as small as 200 ns (10 × ω_mod_/2π ~ 50 MHz). It should be noted, however, that other factors may limit measurements at high frequencies (e.g., attenuation of the ac signals transmitted to the cantilever).

Time-resolved measurements are performed by recording curves of the demodulated Fourier components of the time-periodic surface potential (*V_n_*,Φ*_n_*) as a function of the harmonic number (*n*). This is done by sequentially sweeping the output signal from the second channel of the AWG (i.e., *n*ω_mod_ with *n* = 1, *n* = 2, *n* = 3, etc.). To that end, a pre-programmed list of waveforms is played, and the triggering is done by TTL pulses generated by the AFM controller. The AWG waveform playlist is programmed by using the Keysight benchlink pro software, and the TTL pulses are generated during the course of the spectroscopic ramp by using a user-available high speed digital output of the Mimea unit.

To measure the “dc” components of the Fourier spectrum (*V*_0_ and the reference phase Φ_0_), the sideband generation is switched during a dedicated time window within the spectroscopic ramps (i.e., from DHe-KPFM to standard AM-heterodyne KPFM configuration). To do so, a Python routine is used to toggle the output configuration of the HF2LI modulation module (see Supporting Information File 1), as a function of a trigger signal sent by the Mimea unit. The full phase spectrum (Φ*_n_*) is eventually reconstructed by shifting the data set from Φ_0_ (n_4 sidebands) or Φ_0_ + 180° (n_1 sidebands).

An external fibre-coupled laser source (Omicron Laserage, PhoxX+ at 515 nm) was used for sample illumination through an optical viewport of the UHV AFM chamber. Samples can be illuminated either from the front or from the back using sample holders with on-board mirrors [3]. The latter configuration is used in the case of organic BHJ thin films (processed on transparent substrates, i.e., glass coated with indium thin oxide). The light intensity is adjusted using the laser power control and optical density filters. In the following, for each measurement, the optical power *P*_opt_ (maximum pulse intensity in the case of experiments under modulated illumination) is given in the corresponding figure caption. The *P*_opt_ value is roughly defined per unit area, taking into account the diameter of the laser beam (approximately 1 mm) and the angle of the optical beam with respect to the plane of the substrate.

## Results and Discussion

### Dual-heterodyne Kelvin probe force microscopy under electrical pumping: benchmarking

**Figure 2:**
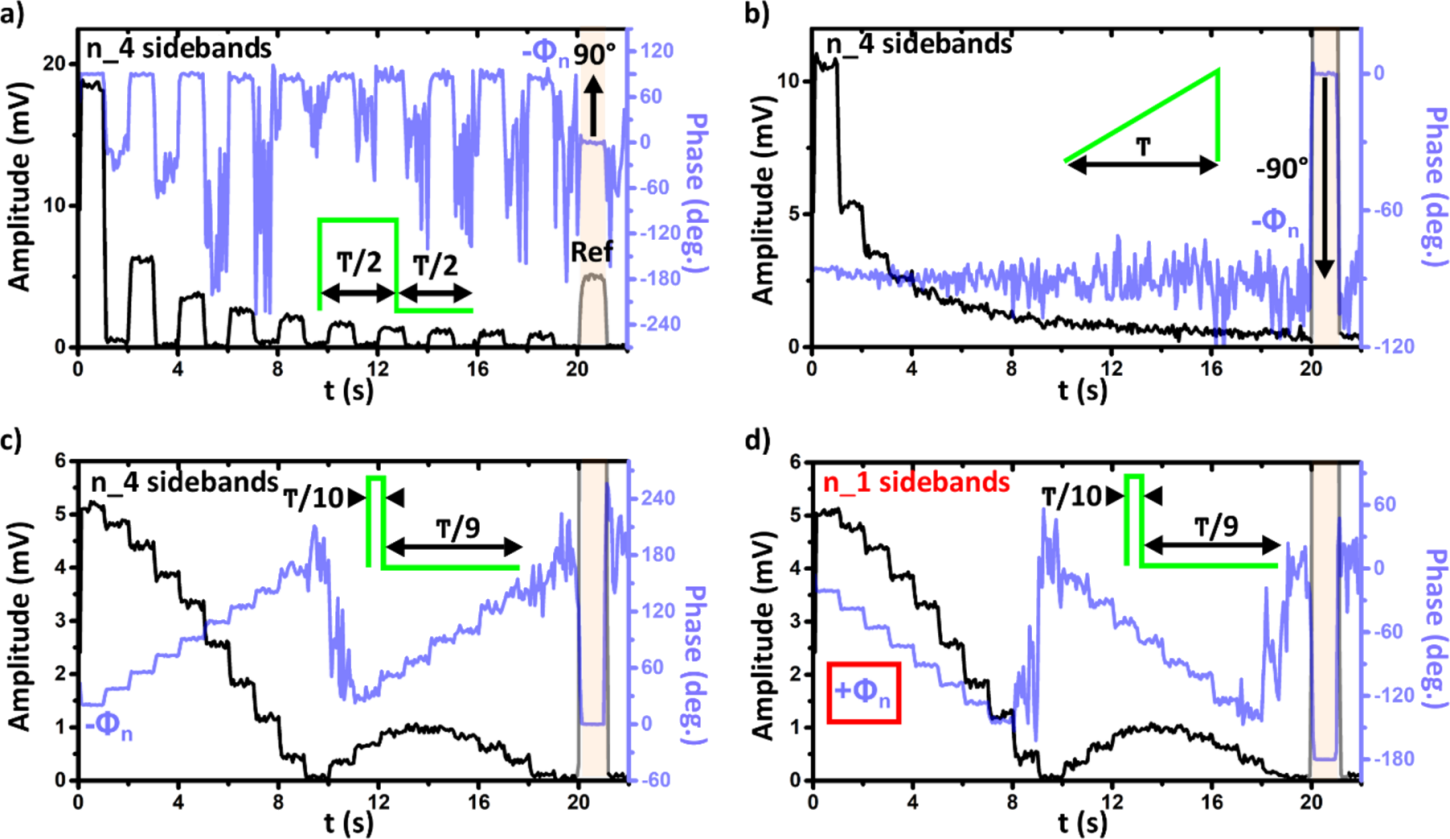
Benchmarking of DHe-KPFM under electrical pumping. Plots of the demodulated amplitude and phase (lock-in outputs) at the second cantilever eigenmode as a function of time, recorded on an HOPG sample under electrical pumping (pump period *T* = 100 µs and peak-to-peak amplitude of 1 V for all spectra). ω_0_/2π ≈ 83.3 kHz, ω_1_/2π ≈ 519.3 kHz, Δω_0_/2π = −25 Hz, ω_mod_/2π = 10 kHz, *U*_ac_ = 0.5 V. The first 20 harmonics have been recorded (*n* = 1 to 20, 1 s per harmonic). a) Square-wave pump with a 50% duty ratio. b) Saw-tooth ramp. c,d) Square-wave pump with a 10% duty ratio. a,b,c) Rejection at ω_1_ of the n_4 sidebands, ω_ac_ = ω_1_ – (ω_0_ + Δω_0_) + *n*ω_mod_. d) Rejection at ω_1_ of the n_1 sidebands, (ω_ac_ = ω_1_ – (ω_0_ + Δω_0_) − *n*ω_mod_). The controller configuration is switched in standard AM-heterodyne KPFM (ω_ac_ = ω_1_ – (ω_0_ + Δω_0_)) during the 20 s < *t* < 21 s time window (highlighted by a semi-transparent pink area). This allows setting a reference phase level (zero or 180° depending on the set of sidebands used to probe the intermodulation products, see main text).

Similar to the case of pp-KPFM [11], we tested this new implementation by performing DHe-KPFM measurements under electrical pumping on a highly oriented pyrolytic graphite substrate (HOPG). For these tests, the sample was electrically connected to the AWG (channel 1 in Figure 1) by mounting the HOPG substrate onto a sample holder designed with in situ electrical contacts [11]. The results of four measurements with different pump signals are shown in Figure 2. In all cases the pump period was fixed at 100 µs, and the pump shape illustrates a textbook case in the field of Fourier transforms (the Fourier coefficients for all waveforms are derived in Supporting Information File 1).

The results of the first two measurements alone would be sufficient to demonstrate the ability of DHe-KPFM to probe the Fourier spectrum of the time-periodic electrostatic surface potential. The pumps consisted of a square wave with a 50% duty ratio (Figure 2a) and a saw-tooth (Figure 2b). In either case, the amplitude and phase spectra almost perfectly match those expected from a simple Fourier transform of the pump.

In short, the square-wave spectrum has only odd harmonics, while the saw-tooth has all of them. In both cases, the amplitude is inversely proportional to the harmonic number, and the phase shows almost perfect quadrature with respect to the dc reference. This last point already demonstrates the validity of the proposed procedure for an absolute determination of the phase signal.

This conclusion is reinforced by the second test results (Figure 2c and Figure 2d), which have been both carried out with a square wave pump, for which the duty ratio was reduced to 10%. The only difference here is that the second measurement (Figure 2d) has been performed by rejecting the n_1 sidebands at the second eigenmode, instead of the n_4 ones (Figure 2c). Both spectra are identical, but – as expected (Equations 9–12) – the phases are inverted (i.e., −Φ*_n_* instead of +Φ*_n_*) with respect to the pump Fourier transform in the case of a signal demodulation based on the n_4 sidebands. At the exception of the harmonics directly neighbouring the null component (*n* = 10 in the case of a 10% duty ratio pump waveform), the measured phase values match fairly well the ones expected from the pump Fourier transform. For their part, the amplitude spectra are identical, they display the typical cardinal sine (sinc) shape (see Supporting Information File 1) and perfectly match the calculated ones. A figure where all set of data (experimental and calculated) are gathered is provided in Supporting Information File 1 (Supporting Information File 1, Figure S5).

### Dual-heterodyne Kelvin probe force microscopy under optical pumping on an organic solar cell

To further validate the DHe-KPFM implementation, a second set of measurements was performed under optical pumping on an organic photovoltaic donor–acceptor blend. As in our previous works, we used the PTB_7_:PC_71_BM tandem. We refer the reader to our former reports [4,11] and review articles for a detailed introduction to KPFM on organic heterojunctions [20], and to the specificities of the PTB7:PC_71_BM blend.

We limit ourselves here to recall that in these organic blends, charge photogeneration can be understood, in a first approach, as the result of exciton dissociation into Coulomb bound charge transfer (CT) states at the donor–acceptor interfaces. This event is finally followed by the dissociation of the CT states into delocalized carriers of opposite sign on both sides of the D–A interface.

In the context of this work, it is also worth recalling that KPFM investigations are carried out in an "open circuit voltage" configuration. In such a geometry, the surface photovoltage probed under continuous-wave illumination results from a balance between the processes of photogeneration and carrier annihilation (by electron–hole recombination). In addition, from the venture of time-resolved KPFM under pulsed illumination, the SPV dynamics are mainly governed by the effective carrier mobility and trapping mechanisms. The main factor limiting the photocharging dynamics is indeed the effective mobility of holes and electrons (under the effect of drift diffusion) in the donor and acceptor networks. After the application of a light pulse – and during the duration of the pulse – the charge mobility limits the rate at which an electrostatic equilibrium can be reached in the sample. Clearly, this parameter also has an impact on the SPV decay dynamics. However, in the latter case, the slower trap release processes are actually the limiting factor. In particular, in the case of the PTB7:PC_71_BM system, it is now well-established that isolated PC_71_BM clusters act as electron trapping centres. The blend morphology, phase composition and concentration gradients of the donor and acceptor species (through the film thickness) can therefore have a dramatic impact on the SPV decay dynamics probed by KPFM.

**Figure 3:**
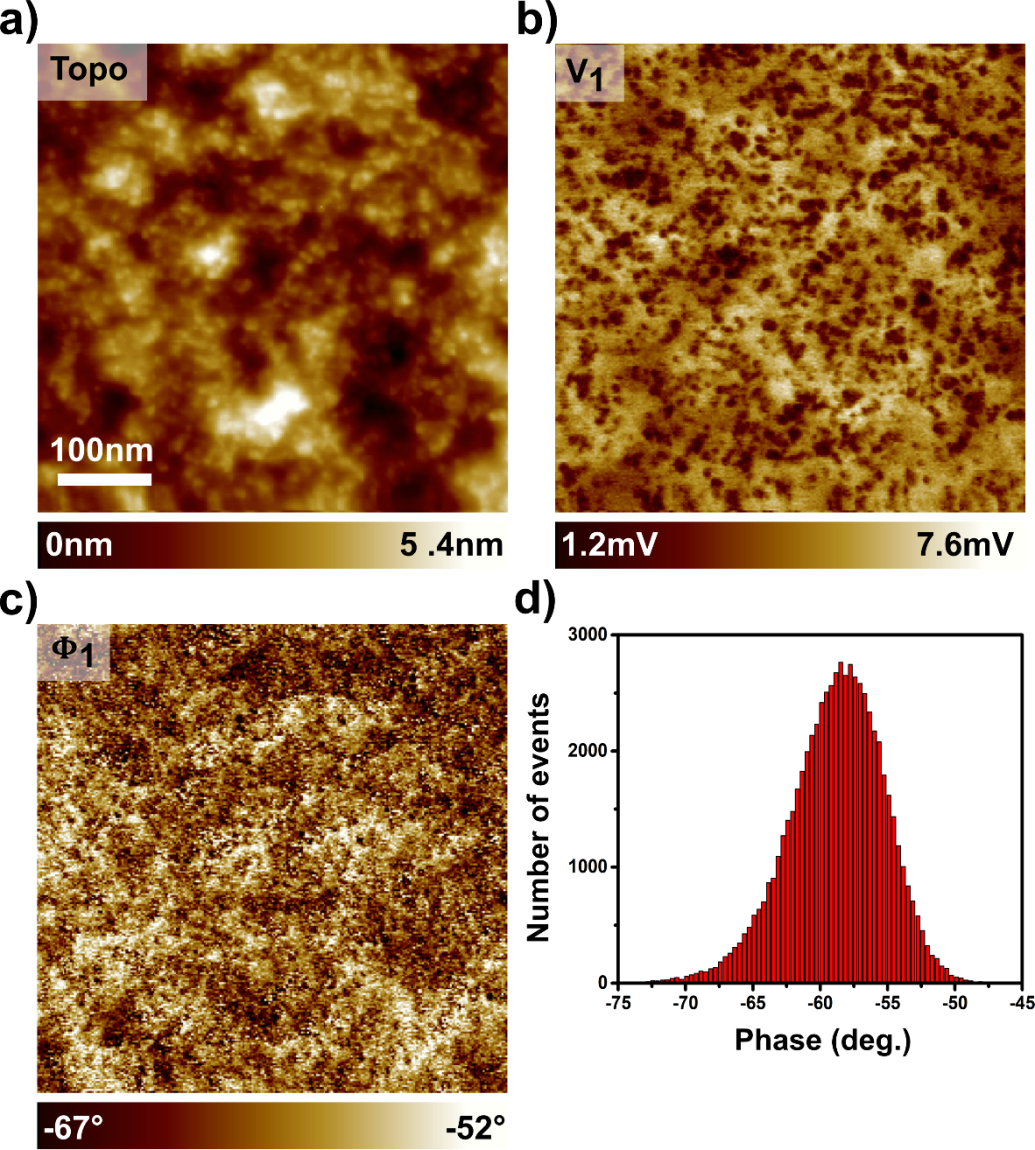
The SPV imaging of an organic solar cell by DHe-KPFM. Topographic (a) and DHe-KPFM (b,c) images (500 × 500 nm, 256 × 256 pixels) of a PTB_7_:PC_71_BM blend acquired under pulsed illumination (λ = 515 nm, *P*_opt_ = 300 mW·cm^−2^, illumination in backside geometry). ω_0_/2π ≈ 83.3 kHz, ω_1_/2π ≈ 519.3 kHz, Δω_0_/2π = −30 Hz, *U*_ac_ = 1 V. ω_ac_ = ω_1_ – (ω_0_ + Δω_0_) + *n*ω_mod_ with *n* = 1. The optical pump consists in a square-shaped signal with a base period of 5 ms (ω_mod_/2π = 200 Hz) and a duty ratio of 20 percent (optical pulse duration: 1 ms). The DHe-KPFM data have been acquired by demodulating (n_4 sideband) the amplitude *V*_1_ (b) and the phase Φ_1_ (c) of the first (*n* = 1) harmonic of the time-periodic SPV. The scan rate was set to 5 s/line. d) Histogram of the phase values.

The sample investigated in this work has been specifically processed to display finely intermixed networks of the donor and acceptor species at the scale of a few tens of nanometres. This was achieved by using diiodooctane as a solvent additive [21], following an identical processing protocol than the one described in our former report [11]. The topographic nc-AFM images (Figure 3a, and numeric zoom in Supporting Information File 1, Figure S6) indicate that the thin-film processing was performed as expected. They display a uniform contrast at the hundred-nanometre scale, indicating that a fine intermixing of donor and acceptor species has been obtained. On a smaller scale (see Supporting Information File 1, Figure S6), nanoscale features appear in the form of small topographic elevations.

The contrasts obtained by imaging the first harmonic components of the time-periodic SPV under modulated/pulsed illumination (amplitude in Figure 3b and phase in Figure 3c) clearly confirm that the phases are segregated at the 10 nm scale. In addition, it is clear that the local topographic protrusions correlate with local minima in the amplitude channel (compare Supporting Information File 1, Figures S6a and S6b).

In all likelihood, the observed contrasts originate from the nanoscale phase segregated donor-acceptor networks. However, it is not possible to perform a phase identification on the sole basis of the harmonic amplitude signal (Figure 3b). Taken alone, that channel gives indeed no information about the polarity of the SPV signal.

In principle, one could use the phase signal for that purpose. Unfortunately, contrary to the case of measurements performed in data-cube spectroscopy (shown hereafter), it is here not possible to determine the phase reference level (Φ_0_). It is still possible, however, to draw a partial conclusion from the examination of the phase data. Their values are indeed distributed in a relatively narrow band, as shown by the histogram in Figure 3d. This indicates that regardless of the area of the sample being scanned, the SPV – or more specifically, its first harmonic component – keeps a constant polarity (otherwise, 180 phase reversals should be observed). As shown hereafter, this assumption is confirmed by spectroscopic DHe-KPFM measurements. It is moreover consistent with the results of complementary AM-heterodyne KPFM measurements of the “static” SPV performed by using a differential spectroscopic protocol. These additional data, presented in Supporting Information File 1 (Figure S7), reveal that the SPV is positive over the entire surface, with values ranging from ca. 40 mV to ca. 170 mV (for 95% of the data according to the SPV histogram, see Supporting Information File 1, Figure S7).

Consequently, the local minima in the DHe-KPFM amplitude image (appearing as dark spots in Figure 3b and Supporting Information File 1, Figure S6b) correspond to areas of the sample where the SPV is "less positive". Thus, from an electrostatic point of view, the most reasonable hypothesis is that these contrasts (and the correlated topographic protrusions) reveal the location of the electron-acceptor (PC_71_BM) sub-network.

These first series of DHe-KPFM measurements has been performed under an optical pump signal featuring a relatively long time-period (5 ms), and 1 ms wide light pulses. This choice of parameters was made according to the results of our previous pp-KPFM measurements on PTB_7_:PC_71_BM blends [4,11], which showed that the SPV decay time constant can vary from tens of µs to several hundreds of µs, depending on the morphology and phase composition. Doing so, we maximized our chances to generate a time-dependent SPV (which first harmonic could be mapped by DHe-KPFM) by choosing a pump time-period higher than a few ms (i.e., the order of magnitude of the longest decay time constants that we observed so far in this system [4]). Naturally, at this stage, the contrasts observed do not allow us to conclude as to the nature of the SPV dynamics (i.e., decay time-constant). In turn, this experiment confirms that DHe-KPFM allows an ac-demodulated imaging of the SPV (to within the *K*_1_ scalar constant) with a very high spatial resolution (a lateral resolution of a few nanometres has been achieved according to image cross-sections, not shown), and at a standard scanning speed (here 5 s/line).

**Figure 4:**
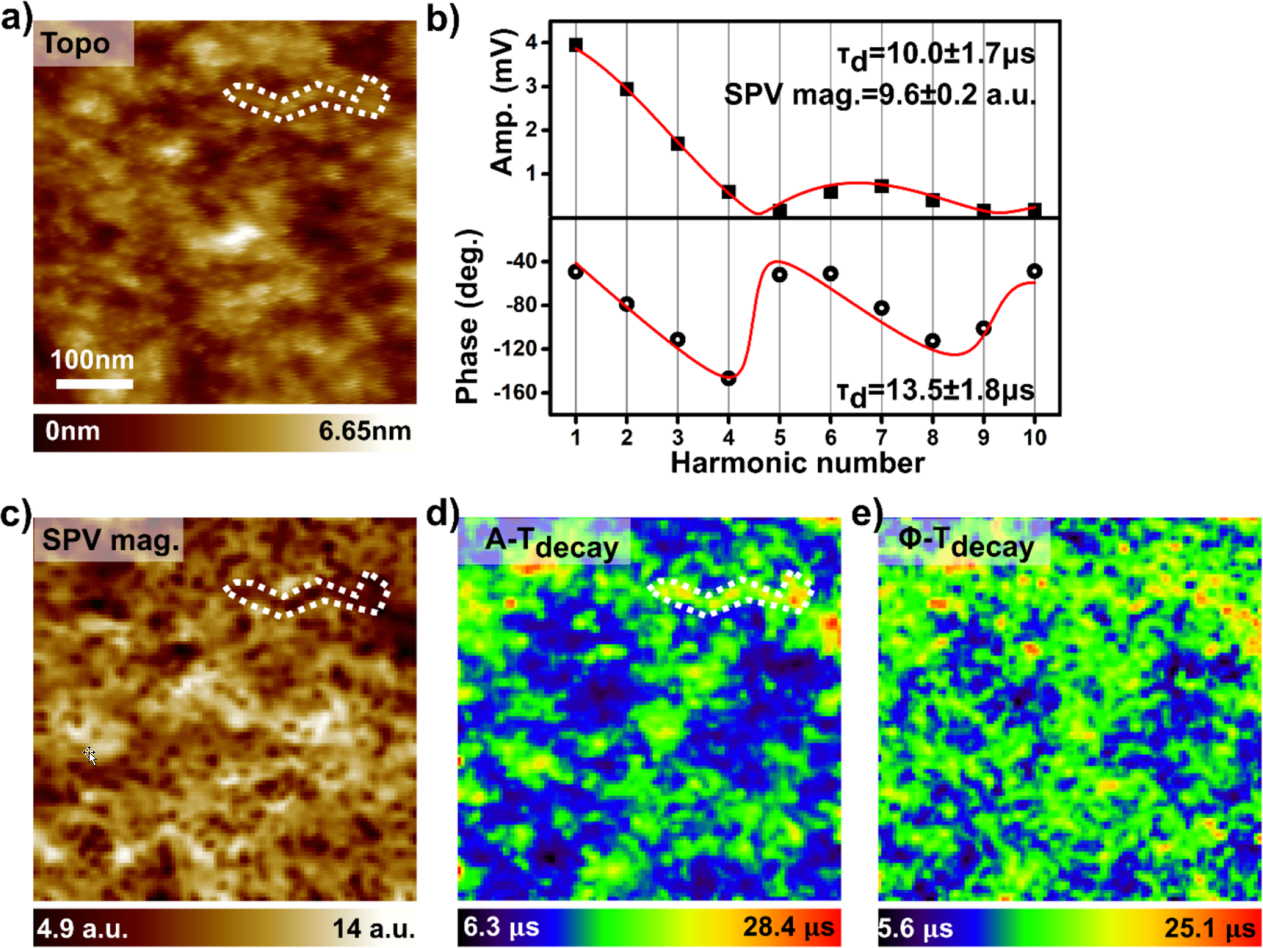
Data-cube DHe-KPFM spectroscopy. Topographic (a) and spectroscopic (b,c,d,e) data acquired onto a 2D grid (102 × 102 pixels) during a data-cube DHe-KPFM measurement on the PTB_7_:PC_71_BM blend. ω_0_/2π ≈ 83.3 kHz, ω_1_/2π ≈ 519.3 kHz, Δω_0_/2π = −30 Hz, ω_ac_ = ω_1_ − (ω_0_ + Δω_0_) + *n*ω_mod_, *U*_ac_ = 1 V. The optical pump consists in a square-shaped signal with a base period of 500 µs (ω_mod_/2π = 2 kHz) and a duty ratio of 20 percent (optical pulse duration: 100 µs). λ = 515 nm, *P*_opt_ = 300 mW·cm^−2^, illumination in backside geometry. a) Sample topography (500 × 500 nm) acquired simultaneously with the spectroscopic data. b) Couple of Fourier spectra (top: demodulated amplitude, bottom: demodulated phase, shifted with respect to the reference Φ_0_, see text) acquired during the data-cube measurement. The curves show the result of adjustments performed by using mathematical functions that are described in the supplementary information (Supporting Information File 1, Equations S18). τ_d_ is a time constant that characterizes the SPV decay dynamics between the light pulses. c,d) Images of the SPV magnitude c) and decay time constant τ_d_ d) recalculated by performing an automated adjustment of the 2D matrix of Fourier amplitude spectra. e) “τ_d_ image” recalculated from the adjustment of the phase data.

It is now time to test the ability of DHe-KPFM to probe the SPV dynamics. Therefore, the 10 first harmonic components (amplitude/phase) were recorded in a data cube spectroscopy experiment under an optical pump, this time operated at a tenfold smaller time scale (100 µs light pulses repeated over a 500 µs period). Figure 4a presents one set of spectra (demodulated amplitude and phase at the second eigenmode as a function of the harmonic number) acquired during this 2D data-cube acquisition. Both the phase and amplitude spectra can be adjusted using fit functions, which are obtained from an analytical calculation of the Fourier components for a time-periodic SPV, whose decay dynamics between light pulses follows an exponential time dependence (with a time constant τ_d_). An initial approach to the problem consists in neglecting the photocharging dynamics (i.e., making the approximation of an instantaneous sample photocharging as soon as a light pulse is applied). The calculation of these functions and of the Fourier coefficients is given in a dedicated section of Supporting Information File 1.

The results of this data-cube DHe-KPFM experiment and of the post-data acquisition data treatment (automated adjustment of the matrix of spectroscopic curves by batch processing with OriginPro software) are presented in Figure 4, along with the topographic image recorded simultaneously pixel by pixel with the spectroscopic curves. Note that the spectroscopic images displayed in this figure have been treated by applying a Gaussian smooth filter. The raw data are shown in Supporting Information File 1, Figure S8, they display the same features, but with a higher noise level. Remember also that the reference phase (Φ_0_) is now measured for each pixel. Accordingly, a post-acquisition correction is applied to the phase data, resulting in absolute values that can be adjusted with the above-mentioned mathematical function (derived from Equation S18 in Supporting Information File 1).

A first sight, both the amplitude and phase data agree reasonably with the calculated Fourier coefficients. The spectroscopic curves (Figure 4b) can be fairly well adjusted, yielding similar SPV time-constant decay values, ranging from a few µs to a few tens of µs (depending on the sample area). In addition to the SPV decay time constant, a second variable parameter – the SPV magnitude (or amplitude in absolute value) – is required to adjust the amplitude (or modulus) coefficients. Following what precedes, the output of that adjustment is given in arbitrary units (since the amplitude coefficients are all probed to within a multiplication by the same unknown factor, *K*_1_).

The calculated SPV magnitude image (Figure 4c) displays contrasts, which definitely confirm our former assumptions. It displays indeed local minima that are correlated with the topographic protrusions. At the same time, the full set of phase data confirms that the SPV polarity remains constant over the entire sample area. Whatever the harmonic, the phase values are indeed distributed in ranges that are incompatible with local polarity inversion (phase images for each harmonic are presented in Supporting Information File 1, Figure S9).

On average, higher decay time constants are observed over the areas associated with the PC_71_BM subnetwork. There are clear correlations between the magnitude minima (Figure 4c) and the decay time constant maxima (Figure 4d and Figure 4e) in several parts of the surface. One of these areas is highlighted by dashed contours in Figure 4c and Figure 4d. This observation is consistent with a well-known fact for fullerene derivative-based heterojunctions: PC_71_BM aggregates act as charge trapping centres. The trap release processes become the limiting factor determining the SPV decay dynamics.

It is also important to note that although both sets of data (amplitude/phase) show good agreement, a better data fit was obtained in the case of the amplitude. In fact, although the relative error (Δτ_d_/τ_d_) is on average slightly smaller for the constants fitted from the phase data (17% instead of 25% for the constants calculated from the amplitude), the fit failed (phase only) for a small fraction of the data (see the images of relative errors and time constant histograms in Supporting Information File 1, Figure S8).

At this stage of our investigations, we have not been able to definitively establish the origin of these differences. A hint can be found by considering the fact that the phase signal obviously shows a higher noise level for the harmonics with the smallest magnitude (e.g., *n* = 5, see the phase image in Supporting Information File 1, Figure S9). The question also arises as to whether a better adjustment could be achieved, by taking into account the photocharging dynamics in the mathematical model used to describe the SPV dynamics.

A first approach consists in checking what the dynamics of the SPV are from the perspective of another technique. To that end, we performed a series of complementary spectroscopic measurements (single data points at selected locations on the surface) by switching the configuration of the setup to perform pump-probe KPFM measurements. We will merely remind that now, the SPV time-measurement proceeds by recording the KPFM compensation potential as a function of a time-delay between the optical pump and the probe. The probe is obtained by restricting the application of the modulation bias to a restricted time window. We refer the reader to our former report [4] for more details; the measurements discussed herein have been realized using exactly the same setup configuration. The pp-KPFM spectra (Figure S10 in Supporting Information File 1) reveal that the SPV decay can be relatively well accounted by a single exponential law, with time-constant values fully consistent with the ones calculated from DHe-KPFM spectra. In addition, the pp-KPFM data show that only a few hundreds of nanoseconds are needed to reach an electrostatic equilibrium once a light pulse has been applied to the sample. This supports the idea that, even if we cannot rule out the possibility of a better fit, neglecting in a first step the photocharging dynamics remains (in the current case) a reasonable approximation. Last, we also note that the pp-KPFM data once again confirm the (positive) SPV polarity.

To conclude this part of the work, it is important to check whether the data obtained for this PTB7:PC_71_BM sample are consistent with the results of our previous investigations on BHJ thin films based on the same donor–acceptor tandem. Considering only the SPV decay dynamics, the current result seems to contradict those of previous investigations on other PTB7:PC_71_BM nanophase-segregated blends. Indeed, previous pp-KPFM studies yielded decay time constant values in the order of hundreds of µs to ms for similar PTB7:PC_71_BM blends processed with diiodooctane additive [4,11]. However, it is important to note that, in contrast to the present case, these earlier series of samples exhibited an overall negative SPV. This shows that although all of these samples are “nanophase segregated”, the morphology of the donor and acceptor subnetworks can show significant variation from batch to batch. In fact, the decay time constant dynamics we observed here are reminiscent of the SPV dynamics we previously observed in PTB7-enriched regions of blends processed without solvent additive [4]. The comparison seems even more pertinent when one considers that these PTB7-enriched areas also showed a positive SPV [4]. Although the following model remains tentative, the picture that seems to emerge is that the BHJs investigated in this work feature nanophase-segregated networks with a higher concentration of the electron-donor polymer (accounting for the positive SPV), and/or a lower density of non-percolating PC_71_BM clusters (delaying the SPV decay less).

### Imaging weak surface photovoltage signals: CsPbBr_3_ nanosheets on HOPG

We have just seen that images of SPV magnitude and dynamics can be obtained with high spatial resolution. In this final section, we will show that DHe-KPFM, thanks to the signal enhancement provided by the second eigenmode, can image weak SPV signals that would otherwise be barely detectable.

To illustrate the capabilities of DHe-KPFM in terms of high sensitivity, we have chosen as a model system an optoelectronic interface that can be obtained by depositing caesium lead halide perovskite nanosheets (NSs) on a highly oriented pyrolytic graphite (HOPG) substrate. Lead halide perovskites have emerged recently as materials with unique optical and electronic properties, such as high absorption coefficients, high defect tolerance, and charge mobility. Due to this, they are very promising for the use in many applications, such as LEDs, solar cells, and photodetectors. Reducing their size down to the nanoscale by synthesizing colloidal nanocrystals in solution can allow high control over the perovskite crystallinity and access to various morphologies. Thus, cubic lead halide perovskite nanocrystals have been widely studied and used for various optoelectronic applications [22–23]. More recently, ultra-thin nanoplatelets or nanosheets (several nm thick and hundreds nm large) with very appealing properties were reported [24]. Due to the 2D quantum confinement in the vertical direction and their smooth surfaces with atomic thickness control, very precise control over the optical gap and exciton energy of NSs can be achieved through chemical synthesis, making them very interesting objects for various studies.

As in previous works [25], the CsPbBr_3_ nanosheets (NSs) synthesized for our investigations display an orthorhombic phase (confirmed by X-ray diffraction characterizations, not shown), a thickness of a few unit cells, and lateral dimensions of a few hundreds of nanometres (the synthesis protocol is described in the Supporting Information File 1). Complementary characterizations were carried out by photoluminescence spectroscopy (Supporting Information File 1, Figure S11), scanning electron microscopy (SEM, Supporting Information File 1, Figure S12), and AFM in ambient conditions (Supporting Information File 1, Figure S13), on a series of samples obtained by depositing the NSs (by spin coating the hexane solution where they are dispersed) on silicon and HOPG substrates (for SEM and AFM). Note that prior to SEM and AFM investigations, the excess of ligands was removed by immersing the substrates in an anhydrous ethyl acetate solution.

**Figure 5:**
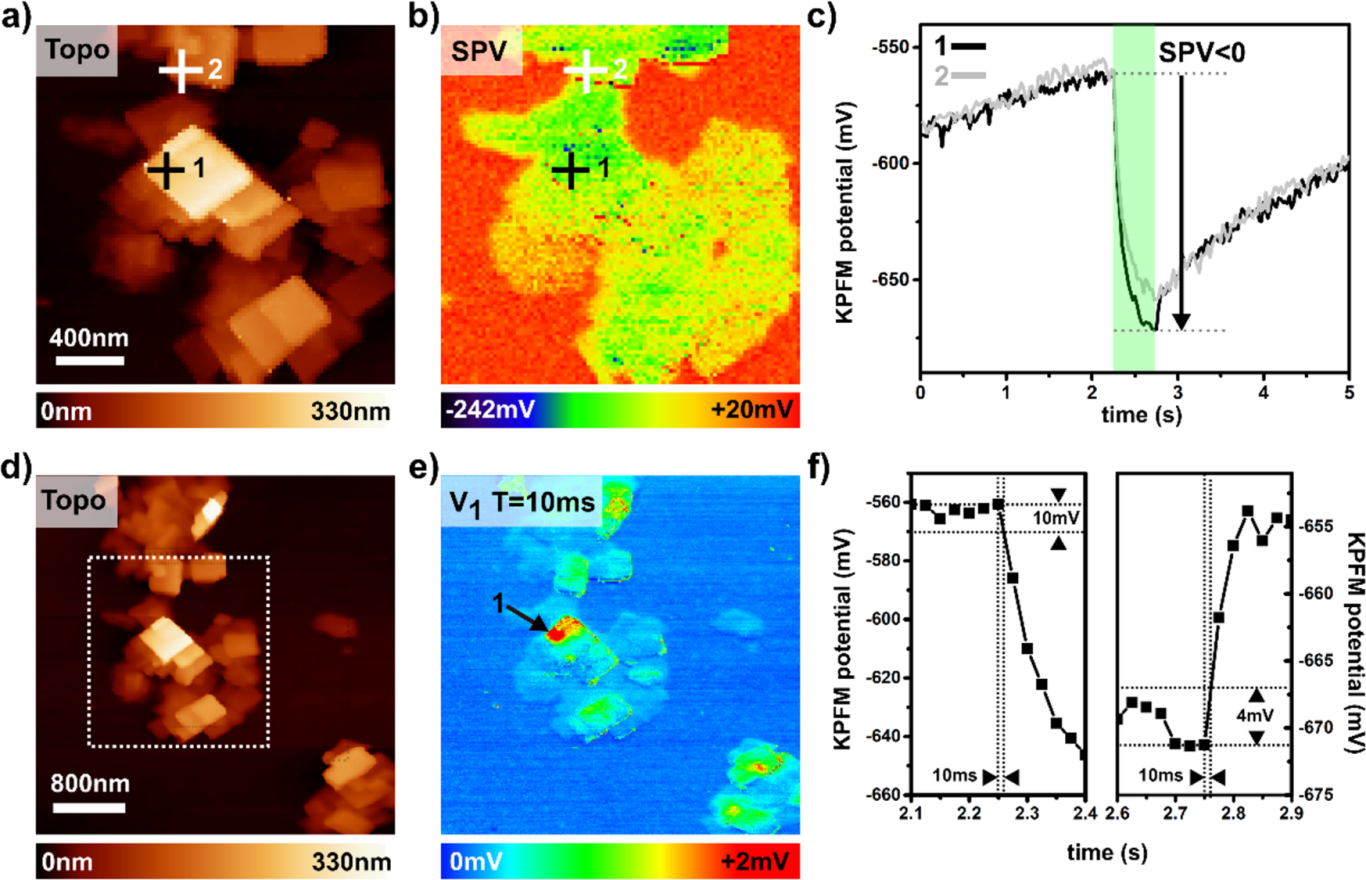
Imaging the photoresponse of a CsPbBr_3_:HOPG interface. (a,b,c,f) Data acquired by differential SPV imaging with the setup configured for AM-heterodyne KPFM (ω_ac_ = ω_1_ − (ω_0_ + Δω_0_)). (d,e) DHe-KPFM measurements (ω_ac_ = ω_1_ − (ω_0_ + Δω_0_) + *n*ω_mod_ with *n* = 1). For all measurements, λ = 515 nm, *P*_opt_ = 1.5 mW·cm^−2^. ω_0_/2π ≈ 63.6 kHz. ω_1_/2π ≈ 401.7 kHz. a,b) Topography a) and SPV b) images (2.1 μm × 2.1 μm). For this measurement, Δω_0_/2π = −4 Hz, ω_ac_ = ω_1_ − (ω_0_ + Δω_0_), *U*_ac_ = 0.5 V. The SPV map is reconstructed from the 2D matrix (96 × 96 pixels) of KPFM(*t*) curves (see text). c) Two curves of the KPFM compensation potential as a function of time (200 pixels), recorded on the locations indicated by markers (labelled 1 and 2) in the topographic and SPV images. For each spectroscopic acquisition, a single 500 ms long light pulse (equivalent to a continuous wave illumination) is applied during the 2.25 s < *t* ≤ 2.75 s time window. Note that an extra 500 ms delay is set between each spectroscopic measurement (i.e., from one image pixel to the next). d,e) Images of the topography d) and amplitude of the first Fourier harmonic e) mapped by DHe-KPFM (256 × 256 pixels, 4 μm × 4 μm). For this measurement, Δω_0_/2π = −12 Hz, ω_ac_ = ω_1_ − (ω_0_ + Δω_0_) + *n*ω_mod_ with *n* = 1, *U*_ac_ = 1 V. The optical pump consists in a square-shaped signal with a base period of 10 ms (ω_mod_/2π = 100 Hz) and a duty ratio of 50 percent (optical pulse duration: 5 ms). The dotted contours in d) delimit approximately the area investigated by differential SPV imaging. The arrow in e) pinpoints the location corresponding to the KPFM(*t*) curve labelled 1 in c). f) Zooms on the KPFM(*t*) curve labelled 1. The aim is to highlight what happens immediately after the light pulse is applied, and after its extinction. Extrapolating the photoresponse to the DHe-KPFM case (modulated illumination with a 10 ms time-period) shows that the modulated SPV magnitude (i.e., *V*_1_, panel e) cannot exceed a few mV.

Sometimes we observe by AFM sample areas where a non-negligible fraction of the surface is covered by NSs monolayers (about 4.5 nm according to the topographic cross section profiles, see Supporting Information File 1, Figure S13). In most cases, however, the NSs form stacks on the surface that are tens to hundreds of nanometres high. This is due to the fact that these objects have a natural tendency to aggregate in solution over time. It is on one of these areas of a HOPG substrate that the DHe-KPFM experiments were performed, the results of which are shown in Figure 5, along with preliminary measurements performed by “differential” SPV imaging (panels a,b,c, and f in Figure 5).

This differential imaging proceeds by recording a 2D matrix of curves of the KPFM potential as a function of time (setup configured in standard AM-heterodyne KPFM, and spectroscopic acquisition performed with an open z-loop). During the spectroscopic ramp, the illumination (continuous wave) is turned on during a pre-determined time window. The difference between the KPFM compensation potential values at the end of the light pulse and just before the light is turned on can be calculated for each pixel.

These differential images (Figure 5b) reveal that a negative SPV develops in the NSs stacks under illumination, with an amplitude exceeding several tens of mV in many places. In a first approach, this negative photocharging may be explained by the existence of a built-in electric field at the interface between the graphite substrate and the CsPbBr_3_ stacks. The KPFM images taken in the "dark state" (Supporting Information File 1, Figure S14) show that the surface potential is in average more positive by ca. 720 mV over the NSs, compared to the bare substrate. A reasonable hypothesis, although not yet definitely confirmed, is that this built-in electric field results from a negative charge transfer mechanism from the CsPbBr_3_ stacks to the substrate. Consequently, the extra charge carriers generated under illumination undergo a drift process, resulting in a spatial separation of electron and hole populations across the space charge region. In other words, the negative SPV that we observe is consistent with a downward band bending at the graphite/CsPbBr_3_ interface. This situation is shown in Supporting Information File 1, Figure S14, together with the "in-dark" KPFM image. While this model seems most plausible, we note that other mechanisms could be invoked to explain the observed SPV (such as illumination-induced band flattening at the top of the stacks, as a result of Fermi level pinning by surface states).

Trapping processes are also clearly involved, as shown by the time course of the KPFM potential (Figure 5c). After each illumination, it takes several seconds for the KPFM potential to return to its initial state (note that an additional 500 ms delay was added between each spectroscopic acquisition, which accounts for the observed difference between the signals at *t* = 0 and *t* = 5 s). Compared to the case of the organic BHJ, the trap release processes now take place on time scales that are several orders of magnitude higher.

A closer examination of the entire set of spectroscopic curves reveals that, in some areas of the sample, a certain fraction of the negative charges may be released more rapidly than in other areas. In fact, some curves exhibit a sharp rise just after the light pulse stops, while others show only a smooth slope increase. Two curves showing this different behaviour, labelled 1 and 2 (corresponding to the locations where they were recorded, as indicated by the markers in Figure 5a and Figure 5b), are shown in Figure 5c.

It turns out that when using DHe-KPFM to map the sample photoresponse under modulated illumination, these specific sample areas become clearly visible. The results of that second data acquisition in DHe-KPFM are presented in Figure 5e (along with the topography in Figure 5d), which displays the amplitude of the first Fourier harmonic (*V*_1_) recorded under a 10 ms periodic square-wave modulated illumination (i.e., a 50% duty ratio). The demodulated signal peaks at exactly the same location (highlighted by an arrow in Figure 5e) where the spectroscopic KPFM(*t*) curves indicate that part of the photopotential is decaying at a faster rate. Again, it is not possible to provide a truly quantitative measurement of the harmonic amplitude *V*_1_ (since the measurement is performed within the multiplicative factor *K*_1_). However, since both series of images were acquired at the same location, a direct comparison can be made between the DHe-KPFM data and the KPFM curves recorded in the time domain.

For that purpose, let us zoom in on the parts of the KPFM(*t*) curves that correspond to the start and end of the “long” light pulses applied for the differential SPV imaging (Figure 5f). These zooms show that, depending on whether one is interested in the SPV growth or decay regime, the electrostatic potential cannot be changed by more than 10 mV or 4–5 mV in a 10 ms time-lapse. Thus, under modulated illumination, the “modulated SPV” magnitude is limited by the slower decay dynamics, and cannot exceed a few mV. The 4–5 mV value appears really as a maximum, since instead of a 10 ms timescale, it would be more rigorous to consider only the 5 ms periods corresponding to the interval between the light pulses used for the DHe-KPFM experiment.

The very high sensitivity of DHe-KPFM is fully demonstrated by the simple fact that clear contrasts are also observed even on sample areas where the magnitude of the modulated SPV is smaller. According to the comparison with the time domain spectroscopic data corresponding to the sample location labelled 2, a resolution at the mV limit may have been achieved. Further investigations would be needed to precisely quantify the magnitude of the modulated SPV component. At the very least, complementary measurements performed under electrical pumping (see Supporting Information File 1, Figure S15) confirm that modulated components as small as a few mV can be detected.

## Conclusion

We have introduced DHe-KPFM as a new approach to measure the Fourier spectrum (or intermodulation products) of a time-periodic surface potential with an atomic force microscope. It has been specially designed for experiments with an nc-AFM under UHV conditions, where the high resonance quality factors of the cantilever are an obstacle to the application of a direct intermodulation scheme [16]. Through a series of experiments on model systems, we have demonstrated the ability of DHe-KPFM not only to probe the Fourier harmonics under optical or electrical pumping, but also to detect modulated components within the resolution limit of other reported KPFM methods. This was made possible by exploiting the amplification of the signal by the second eigenmode of the cantilever. In this sense, DHe-KPFM is the first step towards an open-loop generalization of the AM-heterodyne KPFM method. The only drawback – at this stage – is that the measurement of the amplitude spectrum is achieved within a multiplication by a scalar constant. We have good reason to believe that this issue will soon be solved. Preliminary tests have indeed shown that it is also possible to demodulate other series of sidebands through the second eigenmode, which yields access to the capacitance gradient harmonics. In the short term, it will thus be possible to apply a fully automated retro-conversion process to reconstruct truly quantitative (photo)-potential data in the time domain. This will also provide a simple path for dual-harmonic open-loop KPFM measurements [19], without the need to calibrate the transfer function of the system (keeping in mind that whatever the sideband is, the signal is detected at the fixed second eigenmode frequency).

To conclude this work, we would like to emphasize that beyond time-resolved measurements, perhaps the greatest contributions of DHe-KPFM will come from its ability to detect very weak photomodulated signals. In some cases, this may make it possible to reveal phenomena that would otherwise remain inaccessible to other KPFM-based SPV imaging modes. This applies to any type of system where the net density of spatially separated photogenerated charges is insufficient to generate an electrostatic potential difference greater than a few mV. This situation may for instance occur in some cases in hybrid perovskite thin films when the internal electric fields at the interfaces (with the substrate or the vacuum) are too weak. This could also be the case for molecular heterojunctions in the single layer limit.

## Supporting Information

Fourier coefficient calculation and derivation of the formulas used to fit the spectroscopic DHe-KPFM data acquired under optical pumping on the organic BHJ solar cell (text and Figures S1, S2 and S3). Schematic illustration of the dual frequency mixing effect (Figure S4). Python routine for switching the demodulation configuration. Comparison between the data acquired under electrical pumping with n_4 sidebands, n_1 sidebands, and calculated Fourier coefficients (Figure S5). Numeric zooms from the images acquired by DHe-KPFM on the PTB7:PC_71_BM blend (Figure S6). Complementary measurements on the PTB7:PC_71_BM blend by differential SPV spectroscopy (Figure S7). DHe-KPFM data-cube spectroscopy on the PTB7:PC_71_BM blend: unfiltered (raw-data) images and error images (Figure S8). Images of the first ten harmonic phase signals recorded on the PTB7:PC_71_BM blend (Figure S9). Complementary measurements on the PTB7:PC_71_BM blend by pump-probe KPFM (Figure S10). CsPbBr_3_ nanosheets: synthesis protocol (text); CsPbBr_3_ nanosheets: photoluminescence spectroscopy (Figure S11). CsPbBr_3_ nanosheets: complementary characterization by SEM imaging (Figure S12). CsPbBr_3_ nanosheets: complementary characterization, by “tapping-mode” AFM imaging (Figure S13). CsPbBr_3_ nanosheets on HOPG: in dark KPFM data and built-in interface electric field model (Figure S14); detecting weak modulated components: complementary DHe-KPFM measurements under electrical pump (Figure S15).

File 1 Supporting information.
