# Supplementary material for: Dual-heterodyne Kelvin probe force microscopy
Source: Beilstein J Nanotechnol. 2023 Nov 7;14:1068–84. doi: 10.3762/bjnano.14.88 (PMC10644032; doi:10.3762/bjnano.14.88)
Supplement: File 1 — Supporting information. [file Beilstein_J_Nanotechnol-14-1068-s001.pdf]

BEILSTEIN JOURNAL OF NANOTECHNOLOGY

Supporting Information

for

Dual-heterodyne Kelvin probe force microscopy

Benjamin Grévin, Fatima Husainy, Dmitry Aldakov and Cyril Aumaître

Beilstein J. Nanotechnol. **2023,** *14,* 1068–1084. doi:10.3762/bjnano.14.88

Supporting information

License and Terms: This is a supporting information file under the terms of the Creative Commons Attribution License (https://creativecommons.org/licenses/by/4.0). Please note that the reuse, redistribution and reproduction in particular requires that the author(s) and source are credited and that individual graphics may be subject to special legal provisions.
The license is subject to the *Beilstein Journal of Nanotechnology* terms and conditions: (https://www.beilstein-journals.org/bjnano/terms)

Fourier coefficient calculations

In the following, we will derive the Fourier coefficients used to fit the spectroscopic data obtained on the PTB_7-$PC_{71}BM$ organic photovoltaic heterojunction. First, we recall what the Fourier coefficients are for two basic time-periodic functions, *i.e.*, a pulse train and a sawtooth waveform. These functions correspond to the pump signals used for benchmarking DHe-KPFM under electrical pumping on HOPG.

We consider a time-periodic function *F(t)*, with period $T=1/f=2\pi/\omega$. It can be described by a Fourier series:

$$F(t) = c_0 + \sum_{n\geq 1} |c_n| \cos(n\omega t + \Phi_n) \quad Eq.S1$$

The first term (c_0) represents the time-averaged value of *F(t)* over one period. In the following, we will focus only on the harmonic terms (*i.e.,* $n\geq 1$). The modulus and phase coefficients ($|c_n|$ and Φ_n, respectively) can be calculated using the following equations (in the following, *j* is the imaginary unit and c_n are the complex Fourier coefficients, we refer the reader to basic textbooks for more details):

$$c_n = \frac{1}{T}\int_0^T F(t) e^{-jn\omega t} dt \quad Eq.S2a$$

$$a_n = c_n + c_{-n} \quad Eq.S2b$$

$$b_n = j(c_n - c_{-n}) \quad Eq.S2c$$

$$|c_n| = \sqrt{{a_n}^2 + {b_n}^2} \quad Eq.S2d$$

$$\Phi_n = -atan\left(\frac{b_n}{a_n}\right) \quad Eq.S2e$$

i) Periodic pulse train

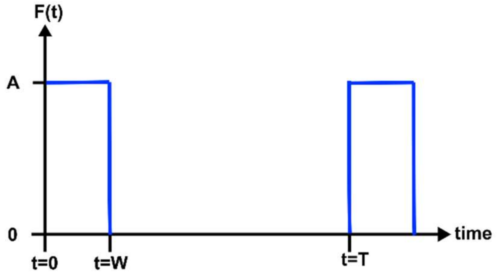

Figure S1 Periodic pulse train of period T, consisting of rectangular pulses of duration W and amplitude A. The duty cycle η is defined by W/T.

In this case, *F(t)* can be defined by:

$$F(t) = \begin{cases} A, & 0 \le t \le W \\ 0, & W < t \le T \end{cases} \quad Eq.S3$$

Here *W* is the pulse width. The duty cycle ratio η is equal to *W*/*T*. Note that the pulse amplitude (or magnitude), labeled *A*, should not be confused with the modulus coefficients of the Fourier series. Confusion may arise from the fact that the $|c_n|$ together are referred to as the signal amplitude spectrum. Note also that we have chosen to use a function with a zero base level for simplicity. The same Fourier coefficients would be obtained for n≥1 if the function were shifted by a DC component. Both comments apply to the following.

The set of equations in S2 yields:

$$c_n = \frac{A}{T}\int_0^W e^{-jn\omega t}dt \quad Eq.S4a$$

$$c_n = \frac{jA}{2\pi n}\left[e^{-jn\omega W} - 1\right] \quad Eq.S4b$$

$$a_n = \frac{A}{\pi n}\sin(2\pi n \times \eta) \quad Eq.S4c$$

$$b_n = \frac{A}{\pi n}[1 - \cos(2\pi n \times \eta)] \quad Eq.S4d$$

$$|c_n| = \frac{A}{\pi n}\sqrt{2(1 - \cos(2\pi n \times \eta)} = 2\eta|\mathrm{sinc}(\pi n \times \eta| \quad Eq.S4e$$

In the case of a square-wave signal (η=0.5), even component are zero, and odd ones are:

$$a_n = 0\,, b_n = \frac{2A}{\pi n}, \quad |c_n| = \frac{2A}{\pi n}\,, \Phi_n \;\; = -90° \;\; (n\; odd) \quad Eq.S5$$

ii) Sawtooth waveform

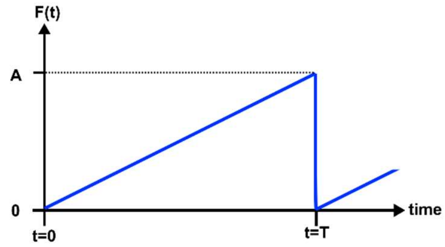

Figure S2 Sawtooth waveform of period T, and amplitude A.

In this case, *F(t)* is defined by:

$$F(t) = \frac{A \times t}{T} \quad Eq.S6$$

$$c_n = \frac{A}{T}\int_0^T t \times e^{-jn\omega t} dt \quad Eq.S7$$

Performing an integration by parts yields:

$$c_n = \frac{A}{T}\left[\frac{-t \times e^{-jn\omega t}}{jn\omega}\right]_0^{\mathrm{T}} + \frac{A}{T}\int_0^{\mathrm{T}} \frac{e^{-jn\omega t}}{jn\omega} dt \quad Eq.S8$$

Which simplifies in:

$$c_n = -\frac{A}{jn\omega} \quad Eq.S9$$

Finally, one gets:

$$a_n = 0\,, b_n = -\frac{A}{\pi n}\,, |c_n| = \frac{A}{\pi n}\,, \Phi_n \;\; = +90° \quad Eq.S10$$

ii) Pulse followed by an exponential decay

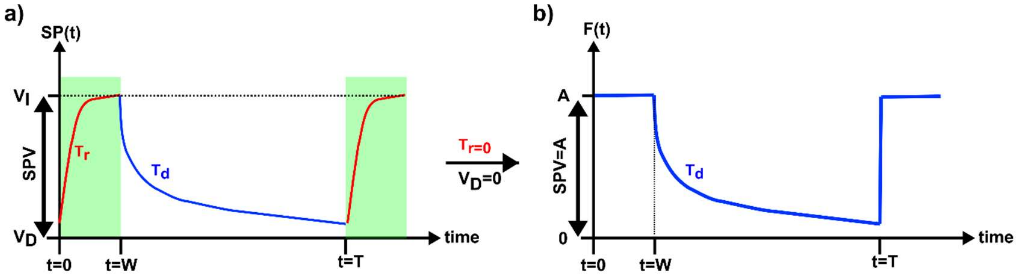

Figure S3 a) Time evolution of the surface potential (SP) under pulsed illumination. The time intervals corresponding to the pump signal are marked by semi-transparent green rectangles. Illumination is performed for $0 \le t \le W$. T: Pump signal period. V_D: surface potential in the dark. V_I: maximum value of the surface potential during illumination. If the pulse duration exceeds the photovoltage build-up or rise time constant (τ_b or τ_r), V_I is equal to the SP value that would be measured under continuous wave illumination (V_{cw}). The "static" (or steady state) surface photovoltage is equal to V_I-V_D. τ_d: surface photovoltage decay time constant. **b)** Time function F(t) (or waveform) used to calculate the Fourier coefficients describing the time-periodic surface photovoltage. We make the approximation of an instantaneous sample photo-charging (*i.e.*, τ_r=0). The waveform amplitude is equal to the steady state SPV, but the base level has been set to zero (this shift does not affect the coefficients for n≥1).

This waveform is used to adjust the spectroscopic data obtained on the organic heterojunction thin film. The sample is exposed to a periodic light pulse train (periodicity T, pulse width W). We assume that the surface photovoltage (SPV) is constant during the pulse duration (approximating an instantaneous photo-charging once the light is turned on, *i.e.* a SPV rise time constant τ_r equal to zero) and that it follows an exponential decay characterized by a single time constant (τ_d) between light pulses. As above, since we only want to calculate the harmonic coefficients (n≥1), we are in the simplified case of a signal characterized by a zero baseline and an amplitude (or magnitude) labeled A.

$$SPV(t) = F(t) = \begin{cases} A, & 0 \le t \le W \\ A \times e^{-(t-W)/\tau_d}, & W < t \le T \end{cases} \quad Eq.S11$$

$$c_n = c_n^p + c_n^d = \frac{A}{T}\int_0^W e^{-jn} \quad dt + \frac{A}{T}\int_W^T e^{-(t-W)/\tau_d} \times e^{-jn\omega t} dt \quad Eq.S12$$

The first integral (c_n^p for "pulse part") has already been calculated above, it corresponds to the case of a pulse train. The second integral (c_n^d for "decay part") can be derived as follow:

$$c_n^d = \frac{A \times e^{W/\tau}}{T} \int_W^T e^{-t(1+jn\omega\tau)/\tau_d} dt \quad Eq.S13$$

$$c_n^d = \frac{A \times \tau_d/T \times (1 - jn\omega\tau_d)}{1 + n^2\omega^2{\tau_d}^2} \left[\cos(n\omega W) - e^{-(T-W)/\tau_d} - j\sin(n\omega W)\right] \quad Eq.S14$$

These complex coefficients can be used to calculate "intermediate" and a_n^d and b_n^d terms, which are added to those already calculated for the pulse part (by the linearity of the Fourier transform). For this purpose, it can be noted that the complex coefficients can be described by a sum of a functions, respectively even and odd in n. This facilitates the calculation of a_n^d and b_n^d (following Eqs. S2b and S2c).

$$c_n^d = \frac{A \times \tau_d/T}{1 + n^2\omega^2{\tau_d}^2} [Even(n) - jOdd(n)] \quad Eq.S15$$

$$Even(n) = \left[(\cos(n\omega W) - e^{-(T-W)/\tau_d}) - n\omega\tau_d \times \sin(n\omega W)\right] \quad Eq.S16a$$

$$Odd(n) = \left[\sin(n\omega W) + n\omega\tau_d \times (\cos(n\omega W) - e^{-(T-W)/\tau_d})\right] \quad Eq.S16b$$

One gets:

$$a_n^d = \frac{2A \times \tau_d/T}{1 + n^2\omega^2{\tau_d}^2} \left[(\cos(n\omega W) - e^{-(T-W)/\tau_d}) - n\omega\tau_d \times \sin(n\omega W)\right] \quad Eq.S17a$$

$$b_n^d = \frac{2A \times \tau_d/T}{1 + n^2\omega^2{\tau_d}^2} \left[\sin(n\omega W) + n\omega\tau_d \times (\cos(n\omega W) - e^{-(T-W)/\tau_d})\right] \quad Eq.S17b$$

Finally, the total coefficients are:

$$a_n = \frac{A}{\pi n} \sin(2\pi n \times \eta) + \frac{2A \times \tau_d/T}{1 + n^2\omega^2{\tau_d}^2} \left[(\cos(2\pi n \times \eta) - e^{-(T-W)/\tau_d}) - n\omega\tau_d \times \sin(2\pi n \times \eta)\right] \quad Eq.S18a$$

$$b_n = \frac{A}{\pi n} [1 - \cos(2\pi n \times \eta)] + \frac{2A \times \tau_d/T}{1 + n^2\omega^2{\tau_d}^2} \left[\sin(2\pi n \times \eta) + n\omega\tau_d \times (\cos(2\pi n \times \eta) - e^{-(T-W)/\tau_d})\right] Eq.S18b$$

These last coefficients are used to generate analytical formulas for the modulus (using Eq. S2d) and phase (Eq. S2e) coefficients that describe the time-periodic SPV signal. In the latter case (phase), the factor A obviously disappears when the ratio b_n to a_n is made. Only one variable parameter (the decay time constant τ_d) is thus needed to adjust the phase data. Conversely, two parameters (A and τ_d) are used to adjust the amplitude/modulus data (as mentioned above, beware of the possible confusion between the signal amplitude A, and the Fourier amplitude spectrum, *i.e.* the $|c_n|$ coefficients).

Note: to perform the phase adjustment, the function atan2(y,x) must be used instead of atan(y/x). The atan2 function is defined as the argument of the complex number x+jy.

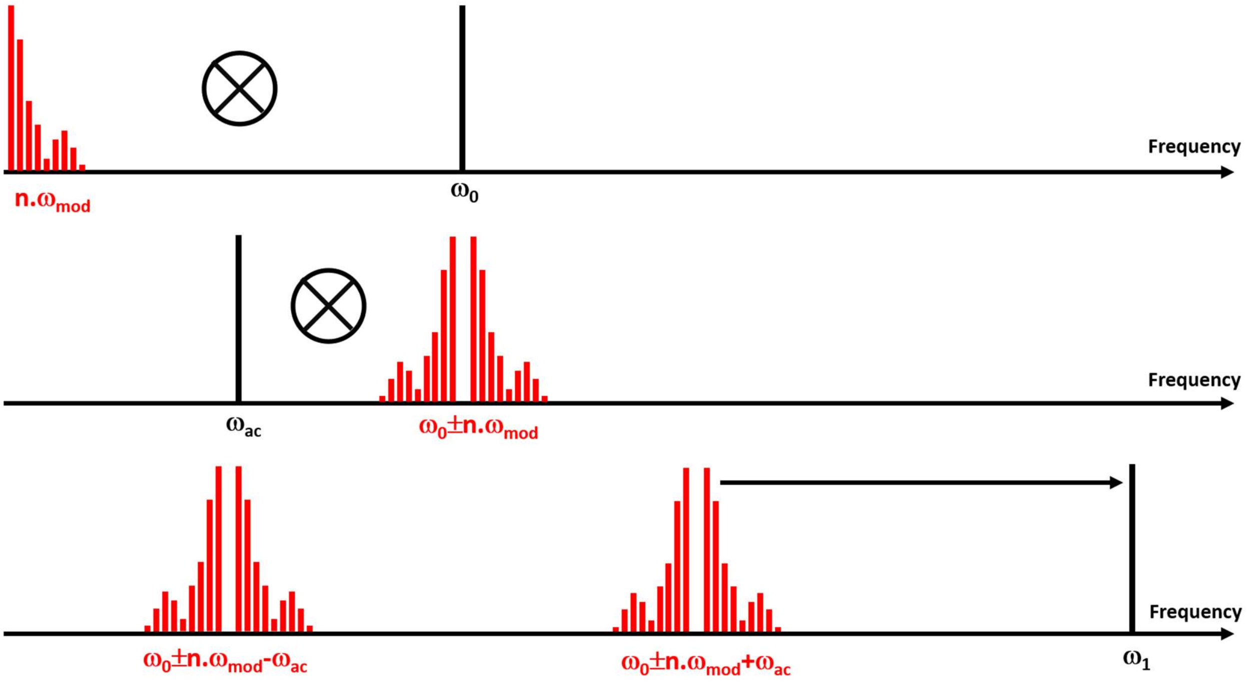

Figure S4 – illustration of the dual frequency mixing effect. **Top panel**: modulated components of the electrostatic potential at harmonic ($n\geq1$) frequencies of the optical pump (ω_{mod}) are depicted in red. NB: here, we made the choice to illustrate a situation where the modulation frequency is set such a way that the frequencies of the first harmonics fall well below the first cantilever eigenmode. This would for instance be the case if $\omega_{mod}/2\pi$=1kHz (while $\omega_0/2\pi$=80kHz). A "first" frequency mixing effect is symbolized (multiplication symbol within a circle) between the cantilever mechanical oscillation (first eigenmode, ω_0) and the modulated components at n. ω_{mod}. **Middle panel**: a "second" frequency mixing effect has to be taken into account, between the ac bias modulation and the spectral components at $\omega_0 \pm$n. ω_{mod}. Here again, an arbitrary choice has been made, the situation is hypothetically depicted with a bias modulation frequency falling below ω_0. This has been done for pedagogical purposes only. Note also that there is off course no physical meaning to assume that there are two mixing processes that occur "one after the other", all effects take place simultaneously. **Bottom panel**: 4n side bands result from the dual frequency mixing. By setting an appropriate value for the modulated bias frequency ω_{ac}, one can "transfer" a given side band at the second cantilever eigenmode ω_1.

Python routine for switching the demodulation configuration

The following script can be used to switch the HF2LI configuration during the spectroscopic ramps.

```
import time
t_end = time.time() + val1
while time.time()<t_end:
        sample=daq.getDouble('/dev18005/auxins/0/values/0')
        if sample < val2 and sample > val3 :
        daq.setint('/dev18005/mods/0/enable', 0)
        timesleep(val4)
        daq.setint('/dev18005/mods/0/enable', 1)
```

val1 : is a time-value exceeding the experiment duration.

sample=daq.getDouble('/dev18005/auxins/0/values/0' : samples a signal provided by the SPM controller during the spectroscopic ramp.

if sample < val2 and sample > val3 : val2 and val3 are threshold values that correspond to a certain time-window during the spectroscopic ramp.

daq.setint('/dev18005/mods/0/enable', 0): toggles the HF2LI modulation unit such a way, that it will not combine the reference signal at $\omega 1$-$\omega 0$ with the reference signal at n.ωmod. Instead, the signal at the output of the HF2LI modulation unit simply "follows" the reference signal at ω_1-ω_0 provided by the MFLI.

val4 : time-value that defines the time-window during which the demodulation chain is operated in standard AM-heterodyne KPFM configuration.

daq.setint('/dev18005/mods/0/enable', 1): toggles back the HF2LI unit in DHe-KPFM configuration. Now the HF2LI modulation unit performs a combination of the signals at $\omega 1$-$\omega 0$ and n.ωmod (i.e. generation of a reference side band).

dev18005: HF2LI device

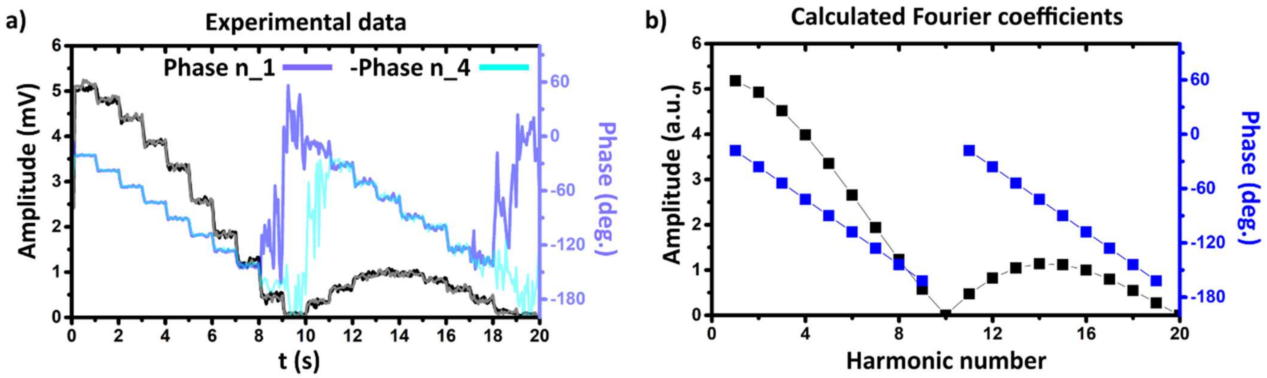

Figure S5 – a) Comparison of DHe-KPFM amplitude/phase spectra acquired by rejecting at the second eigenmode the n_4 side bands ($\omega_{ac}=\omega_1-(\omega_0+\Delta\omega_0)+n.\ \omega_{mod}$) or the n_1 side bands ($\omega_{ac}=\omega_1-(\omega_0+\Delta\omega_0)-n.\ \omega_{mod}$). The data are the same that the ones shown in Figure 2. The phase has been inverted (minus sign) for the first series of data (n_4 side bands). **b)** Plot of the Fourier coefficients obtained by an analytic calculus. The amplitude has been "normalized" (*i.e.* by fixing its value to 5.2 for n=1) to ease the comparison with the experimental data.

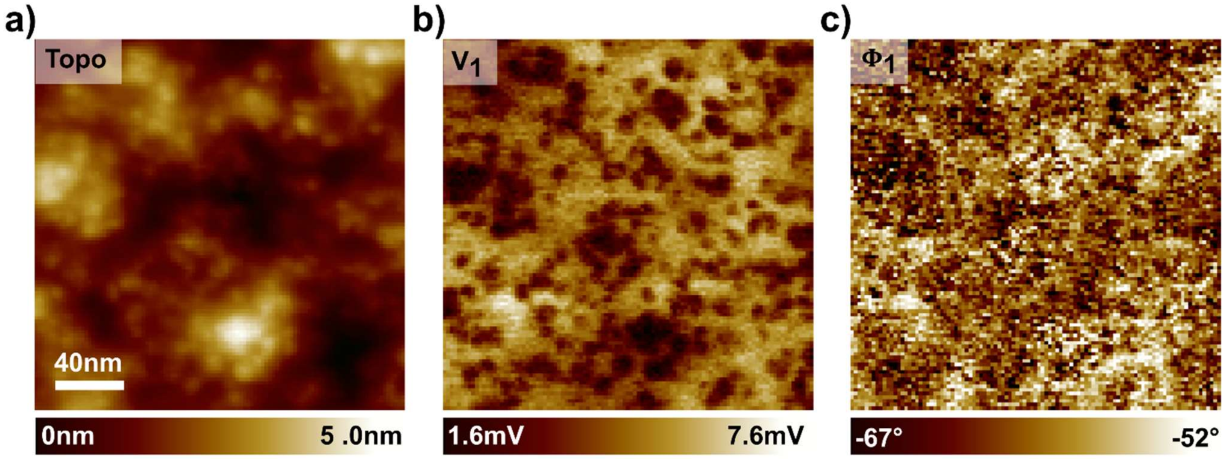

Figure S6 – numeric zooms from the images (Figure 2, main text) acquired by DHe-KPFM on the PTB_7:$PC_{71}BM$ blend. **(a)** Topography **(b,c)** amplitude and phase of the first harmonic. Pulsed illumination (λ=515nm, P_{opt} = 300 mW.cm^{-2}, illumination in backside geometry). $\omega_0/2\pi \approx 83.3$kHz. $\omega_1/2\pi \approx 519.3$kHz. $\Delta\omega_0/2\pi = -30$Hz. $\omega_{ac} = \omega_1 - (\omega_0 + \Delta\omega_0) + n.\omega_{mod}$ with n=1, U_{ac}=1V. The optical pump consist in a square-shaped signal with a base period of 5ms ($\omega_{mod}/2\pi$=200Hz) and a duty ratio of 20 percent (optical pulse duration: 1ms).

.

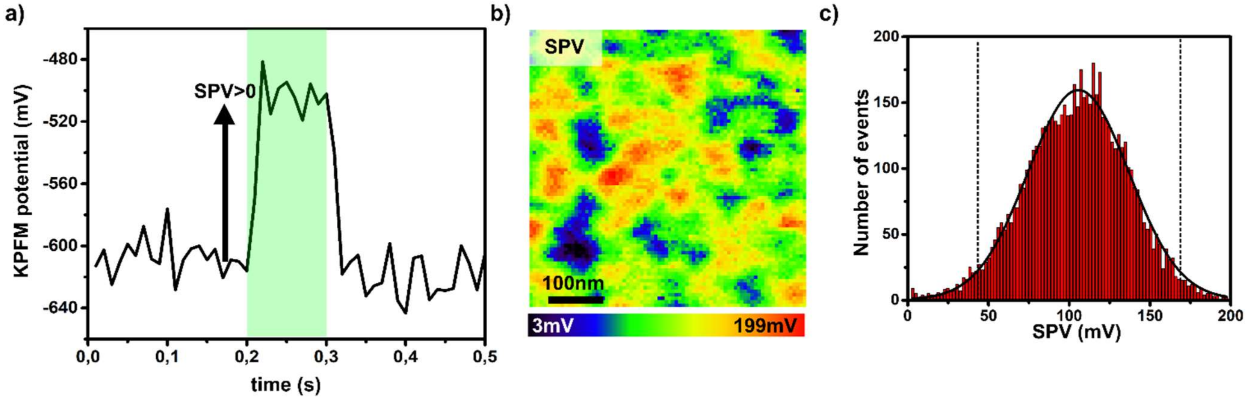

Figure S7 – a) Curve of the KPFM potential as a function of time, recorded over the PTB7:$PC_{71}BM$ blend during a data-cube acquisition. The setup is configured for standard AM-heterodyne KPFM measurements, and the spectroscopic acquisition is performed with an open z-loop. $\omega_0/2\pi \approx 83.3$kHz, $\omega_1/2\pi \approx 519.3$kHz, $\Delta\omega_0/2\pi = -30$Hz, $\omega_{ac} = \omega_1 - (\omega_0 + \Delta\omega_0)$, $U_{ac} = 0.5$V. During the spectroscopic ramp, the illumination (continuous wave) is turned on during a pre-determined time-window. The difference between the KPFM compensation potential values in the dark state and under illumination can be calculated for each pixel. **b)** Differential SPV image calculated from the 2D matrix of spectroscopic data shows that the SPV is in overall positive whatever the sample area. $\lambda = 515$nm, P_{opt} = 300 mW.cm^{-2}, illumination in backside geometry. **c)** Histogram of the SPV values. The curve shows the result of the data adjustment by a single Gaussian peak. The dotted lines indicate the 95% interval (x_c +/-2σ. x_c: peak centre. σ: standard deviation).

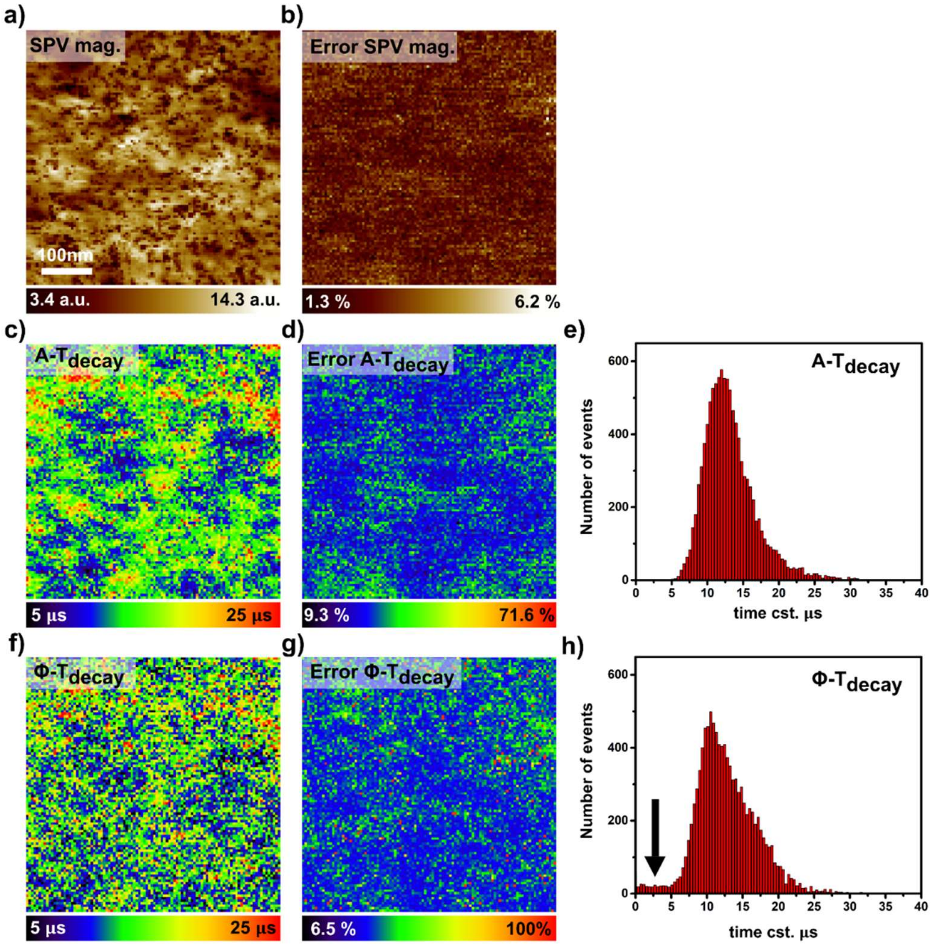

Figure S8 – DHe-KPFM data-cube spectroscopy on the PTB7:$PC_{71}BM$ blend. Images of the SPV magnitude and decay time-constant obtained by performing an automated adjustment of the 2D matrix of spectroscopic curves. This procedure allows also mapping images of the standard (relative) error generated by the fit for each pixel (in percent). Note that the same data are shown in Figure 4 (main text), but in that case the high frequency noise has been removed by applying a Gaussian smooth filter. **a,b)** SPV magnitude a) and standard error ΔSPV/SPV×100 b) recalculated by performing an adjustment of the 2D matrix of amplitude spectra. **c,d,e)** SPV decay time constant τ_d image c), standard error image $\Delta\tau_d/\tau_d$×100 d), and histogram of the decay time constant values e). Results obtained by adjusting the matrix of amplitude spectra. **f,g,h)** SPV decay time constant τ_d image f), standard error image $\Delta\tau_d/\tau_d$×100 g), and histogram of the decay time constant values h). Results obtained by adjusting the matrix of phase spectra.

Concerning the SPV decay time-constant, the fit error is in average slightly smaller in the case of the data calculated by adjusting the phase spectra. Nevertheless, a certain fraction of the phase spectra could not be properly adjusted. For these “bad” pixels, the error diverges. The upper limit of the color scale has thus been fixed to 100%. Errors above that threshold appear as magenta pixels in g). These points correspond to an “anomalous” distribution of time-constant values in the histogram h), highlighted by an arrow.

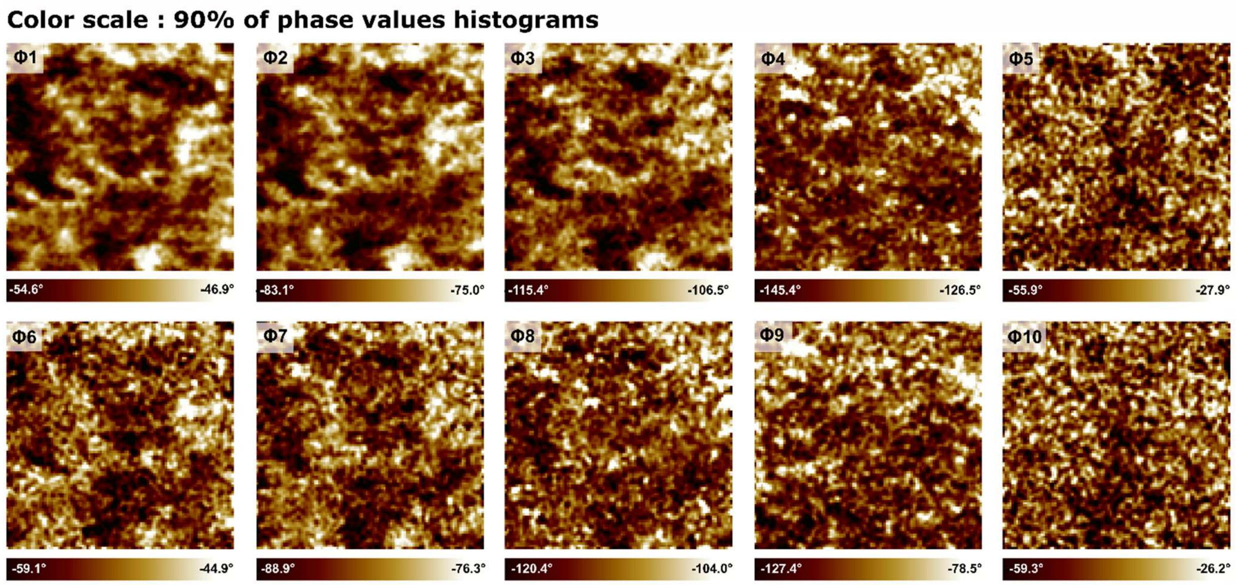

Figure S9 – Images of the first ten harmonic phase signals recorded on the PTB7:$PC_{71}BM$ blend, reconstructed from the matrix of spectroscopic curves (same measurement as the one discussed in Figure 4)

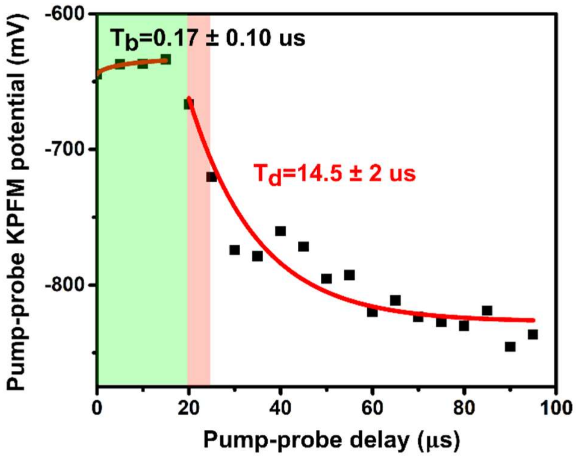

Figure S10 – Curves of the time-resolved potential probed by pp-KPFM as a function of the pump-probe delay. For this measurement the pump and probe duration have been set to 20µs and 5µs, respectively (repetition period: 100µs). Half-transparent green and red rectangles show the respective time-positions of pump and probe signals for a pump-probe delay Δ(t) = 20µs (λ=515nm, P_{opt} = 300 mW.cm^{-2}, illumination in backside geometry). The data corresponding to the photocharging regime and SPV decay regime have been adjusted by functions, that have been derived on the assumption that the SPV time-evolution can be accounted by simple exponential-based functions [S1]. τ_b and τ_d stand for the time-constants that characterize the SPV rise (or build-up) and decay dynamics, respectively. A detailed description of the pp-KPFM setup and data analysis protocol can be found in our previous report [S1].

$CsPbBr_3$ nanosheets: synthesis protocol

The nanosheets were synthesized following the same route as the one described in the work by Shamsi et *al.* [S2]

Preparation of Cesium-oleate (Cs-OA) precursor:

Cs-OA was obtained by loading 0.032 g Cs_2CO_3 with 10 mL oleic acid (OlAc) into a 25 mL 3-neck flask This mixture was dried for 1h at 120 ºC under vacuum, and then heated to 140 ºC under Ar for 30 minutes until all Cs_2CO_3 reacted with OlAc. The major change in this way of preparing Cs-OA precursor is that it is dissolved in OlAc without using ODE, which alone leads to the formation of nanoplatelets (NPls) but not nanocubes (NCs), over a wide temperature range from 50 to 150 °C according to Shamsi *et al.* [S2]

Synthesis of $CsPbBr_3$ nanosheets (NSs):

0.013 g $PbBr_2$ and 10 ml ODE were loaded with 250 µl OA, 250 µl oleylamine (OAm), 250 µl octanoic acid (OctA), and 250 µl octylamine (OctAm) into a 25 ml 3-neck flask and dried under vacuum for 20 minutes at 100 °C. After complete solubilization of the $PbBr_2$ salt, the temperature was increased to 145 °C under Ar and 1 ml of the prepared Cs-OA (heated again at 100 °C) was swiftly injected. Accordingly, we used a volumetric ratio of 0.33 which represents the ratio of short to long ligands. After 5 minutes, the reaction mixture was quenched using a cold water bath [1].

Isolation of $CsPbBr_3$ NSs:

To collect the NSs, 10 ml of anhydrous hexane was added to the crude solution and then the mixture was centrifuged at 700 RPM for 5 minutes. The supernatant was discarded and the NSs were dispersed in 3 ml of hexane.

Purification of $CsPbBr_3$ NSs:

After depositing the solution on HOPG or SiO_2 by spin coating (done at 2000 RPM for 45 seconds), the substrate was gently dipped in anhydrous ethyl acetate (EtOAc) for few seconds to remove the excess of organics and thus be compatible for AFM characterization.

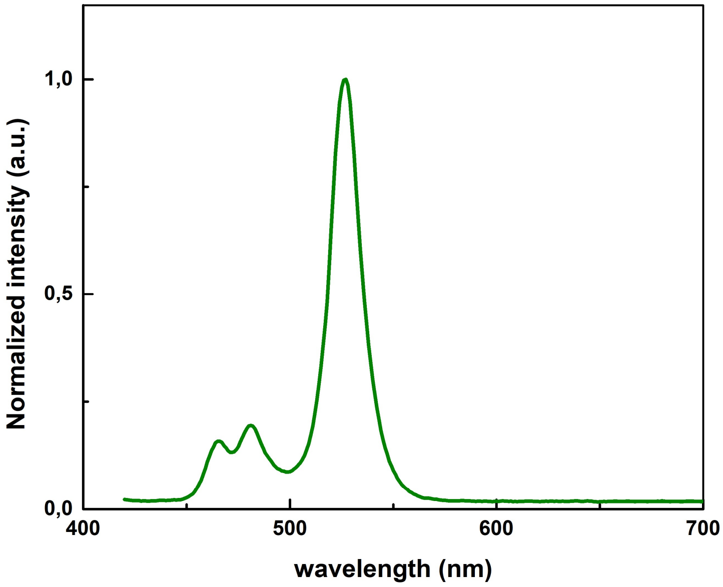

Figure S11 - $CsPbBr_3$ nanosheets : photoluminescence spectroscopy. The main peak is consistent with the existence of nanosheets with a thickness equal or higher than 4-5nm [S2]. The left doublet indicates the existence in the solution of objects (nanosheets) characterized by a smaller thickness. These were not clearly visible in our AFM measurements.

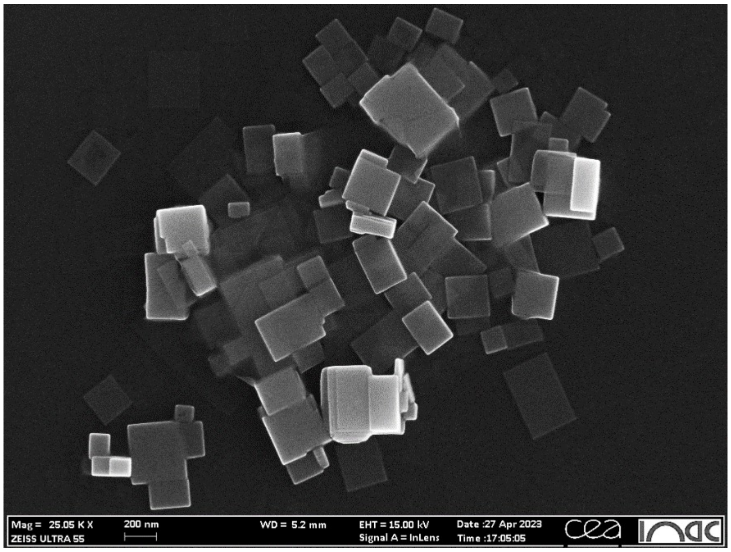

Figure S12 - SEM imaging of $CsPbBr_3$ nanosheets on a silicon substrate (same synthesis batch as the one used for deposition on the HOPG substrate).

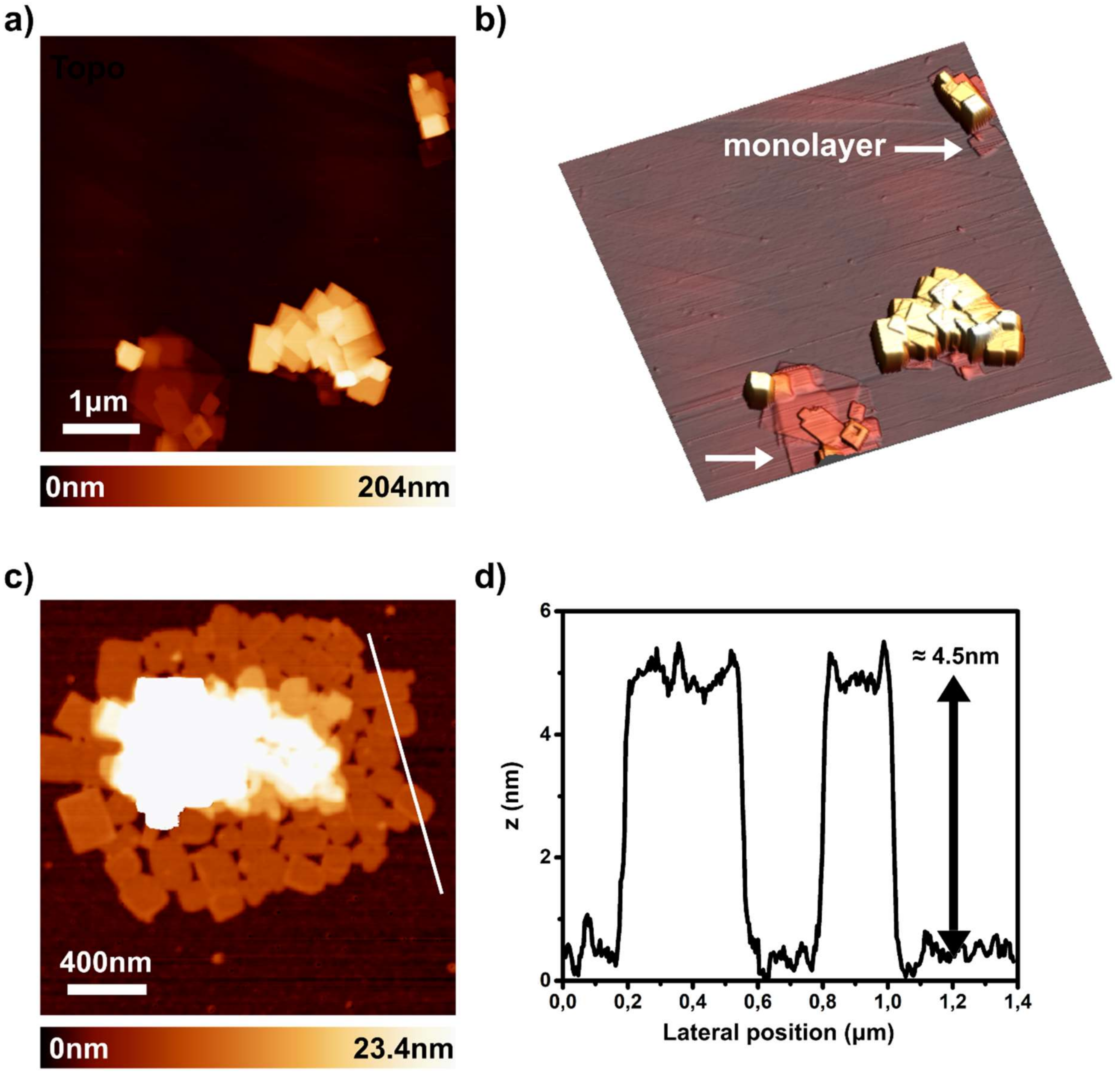

Figure S13 - $CsPbBr_3$ nanosheets: “tapping-mode” AFM characterizations (performed with an ICON setup from Brüker) in ambient conditions. **a,b)** NSs deposited on an HOPG substrate. **a)** Standard topographic representation. **b)** 3D mapping. The arrows pinpoint NSs monolayers. **c,d)** NSs deposited on a silicon substrate. c) Topographic image recorded on an area covered in majority by monolayers. **d)** Topographic profile, corresponding to the path highlighted by a line in c).

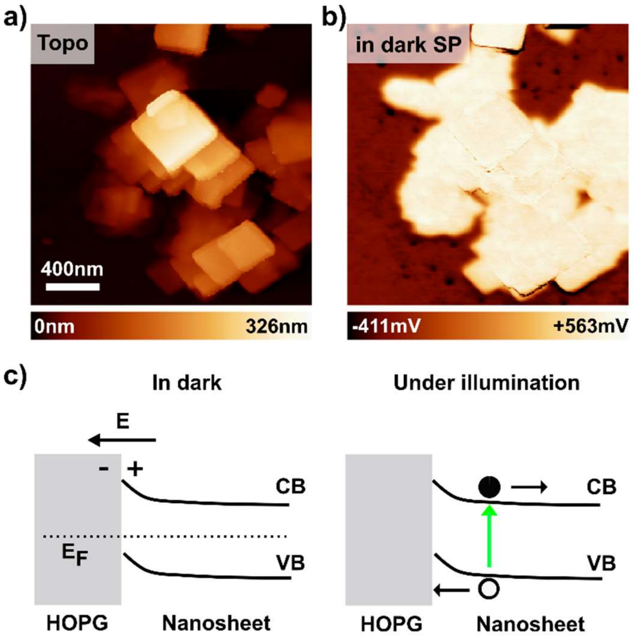

Figure S14 – (a,b) Topography a) and surface potential b) images acquired in "dark conditions" by AM-heterodyne KPFM on the same area than the one investigated by differential SPV imaging and DHe-KPFM (Figure 5, main text). $\omega_0/2\pi \approx 63.6$kHz. $\omega_1/2\pi \approx 401.7$kHz. $\Delta\omega_0/2\pi = -4$Hz. $\omega_{ac} = \omega_1 - (\omega_0 + \Delta\omega_0)$, $U_{ac} = 0.5$V. **c)** Band diagram of the HOPG/$CsPbBr_3$ interface. Under illumination, the build in interface electric field repels the photo-generated electron from the interface region, accounting for the negative SPV.

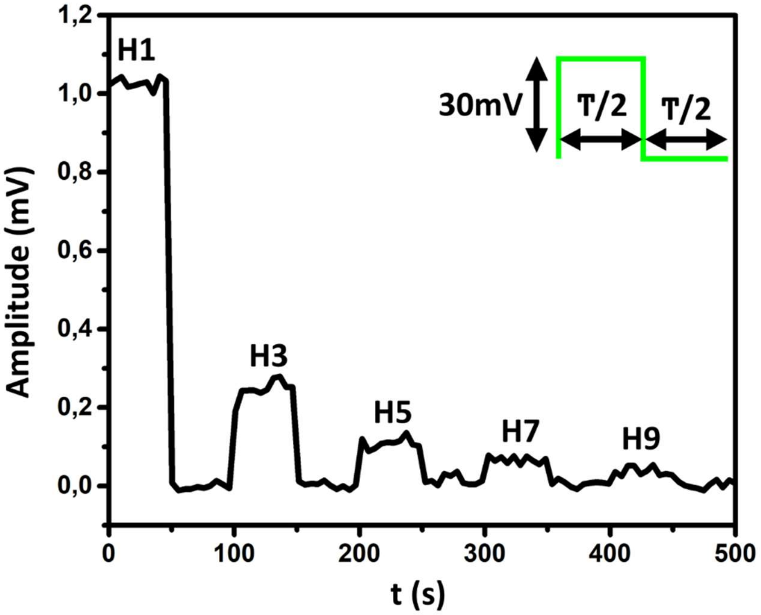

Figure S15 – Plot of the demodulated amplitude (lock-in ouput) at the second cantilever eigemode as a function of time, recorded on an HOPG sample under electrical pumping, with a pump period T=100µs. The pump signal amplitude was set to 30mV peak-to-peak. The first 10 harmonics have been recorded. $\omega_0/2\pi\approx75.4$kHz, $\omega_1/2\pi\approx480.8$kHz, $\Delta\omega_0/2\pi=-12$Hz, $\omega_{mod}/2\pi=10$kHz, $U_{ac}=1$V. According to Equ. S5 (page 4), each odd harmonic amplitude should be equal to $2\times A/\pi\times n$, with A=30mV. The 7th harmonic is clearly visible, demonstrating that modulated components of a few mV only can be detected (with n=7, the amplitude should be equal to 2.7mV)

References

[S1] Aubriet, V.; Courouble, K.; Bardagot, O.; Demadrille, R.; Borowik, Ł.; Grévin, B.

Nanotechnology **2022,** *33,* 225401. doi: 10.1088/1361-6528/ac5542

[S2] Shamsi, J.; Dang, Z.; Bianchini, P.; Canale, C.; Di Stasio, F.; Brescia, R.; Prato, M.; Manna, L*.* *J. Am. Chem. Soc.* **2016,** *138,* 7240-7243. doi: 10.1021/jacs.6b03166.
